# Genomic landscape reveals the dominance of self-catalytic, high-copy group II introns in PMU-deficient complete genomes of PWB phytoplasmas

**DOI:** 10.71150/jm.2511004

**Published:** 2026-03-19

**Authors:** Kiran Kirdat, Malad Mubarak, Pradeep Choudhary, Shivaji Sathe, Amit Yadav

**Affiliations:** 1 BRIC- National Centre for Cell Science, Ganeshkhind, Pune 411007, India; 2 Department of Microbiology, Tuljaram Chaturchand College, Baramati, Maharashtra 413102, India; 3 Ajinkya DY Patil University, Airport Road, Charholi Budruk, Pune 412105, India; 4 Dr. DY Patil Biotechnology & Bioinformatics Institute, Pimpri-Chinchwad 411033, India; 5 Regional Centre for Biotechnology, NCR-Biotech Cluster, Faridabad 121001, India

**Keywords:** phytoplasma, Peanut Witches’ Broom, 16SrII group, OGRI, taxogenomics, group II intron, reductive genomes

## Abstract

Phytoplasmas are wall-less obligate parasites of plants and insects. Several phytoplasma strains within the Peanut Witches’ Broom (PWB; 16SrII) group are associated with significant disease losses across diverse crops and weeds. We present complete, single contig genome assemblies for two Indian parthenium phyllody strains, ‘*Candidatus* Phytoplasma asiaticum’ PR34 and ‘*Ca*. P. australasiaticum’ PR08, generated through host DNA depletion and hybrid Illumina–Nanopore sequencing. Both genomes display characteristic features of reductive evolution (∼614 kb and 589 kb, respectively) but show notable differences from previously sequenced PWB phytoplasmas. In contrast to most of PMU-rich phytoplasma genomes, neither PR34 nor PR08 retains intact Potential Mobile Units. Instead, both harbor numerous open reading frames encoding group II intron reverse transcriptase/ maturase proteins, predominantly of the mitochondrial-like type, with PR34 containing 52 and PR08 28 such loci that together constitute > 4% of each genome. These observations support the hypothesis that intron-associated processes may contribute to genome variability in the absence of canonical PMUs. Comparative analyses support the classification of PR34 as a distinct species within the PWB complex and reveal both conserved Sec-dependent effectors (SAP05, SAP11, and SAP54/PHYL1) and lineage-specific secreted proteins with predicted nuclear localization. Additional retained features include functional *sodA* genes and multiple truncated HlyB-like transporters. Collectively, these high-quality genomes illustrate a genomic configuration in which extensive genome reduction and loss of PMUs coexist with the retention of core virulence factors and an expanded repertoire of group II introns, providing a framework for future investigation of genome plasticity in phytoplasmas.

## Introduction

Phytoplasmas are economically significant bacterial pathogens that cause devastating diseases in plants worldwide, leading to substantial yield losses and reduced agricultural productivity. These wall-less bacteria, classified within the phylum *Mycoplasmatota* (class Mollicutes), are obligate intracellular parasites confined to the phloem tissue of plants and transmitted by phloem-feeding insects. They induce a wide range of characteristic symptoms, such as leaf size reduction, witches’ broom, phyllody, virescence, stunting, and sterility, reflecting severe disruption of normal developmental processes across diverse host plants (Kirdat et al., 2023).

Among the various phytoplasma groups, the Peanut Witches’ Broom (PWB) group has emerged as a major cause of crop and weed infections in tropical and subtropical regions, notably affecting pulses and legumes (Duduk et al., 2018). Weeds such as *Parthenium hysterophorus* serve as persistent reservoirs facilitating transmission by leafhoppers, which play a central role in disease spread (Thorat et al., 2016; Yadav et al., 2015). The wide host range and adaptability of PWB phytoplasmas are likely linked to their highly plastic genomes, which display extensive variation in effector gene repertoires and mobile genetic elements that mediate host interactions and virulence (Oshima et al., 2023). These effectors, often associated with Potential Mobile Units (PMUs), are critical for modulating host physiology and expanding host range, though PMUs themselves are highly dynamic and frequently degraded in reduced genomes (Gan et al., 2025), raising questions about alternative mechanisms of genome variability and adaptation.

Initially, the group was represented by two species: '*Ca*. P. citri' and '*Ca*. P. australasiaticum' (White et al., 1998; Zreik et al., 1995). However, recent advancements in genomic sequencing and molecular phylogenetics have significantly expanded our understanding of this group, leading to the description of additional species and genetic variants (Jardim et al., 2023; Kirdat et al., 2023). Whole-genome sequencing facilitates precise taxonomic classification using Overall Genome Relatedness Index (OGRI)-based comparative taxogenomics (Kirdat et al., 2023). However, obtaining phytoplasma genomes remains challenging due to their unculturable nature, low concentration in plant tissues, and repetitive genomic sequences complicating short-read sequencing assemblies. Currently, 40 complete genome assemblies exist among 282 publicly available phytoplasma genomes (~14%), underscoring ongoing technical challenges in obtaining fully assembled genomes. Traditional enrichment techniques, such as cesium chloride gradient centrifugation and pulsed-field gel electrophoresis (PFGE), are now largely superseded by advanced approaches involving host DNA depletion and immunoprecipitation-based enrichment methods (Kirdat et al., 2021; Tan et al., 2021).

Given the significant economic threat posed by Peanut Witches’ Broom (PWB) phytoplasmas to pulse crops globally, particularly in major producing countries like India, we selected two reference strains, ‘*Ca*. P. asiaticum' strain PR34 and ‘*Ca*. P. australasiaticum’ strain PR08, for comprehensive genomic characterization. This study specifically describes optimized enrichment methods and sequencing strategies used to generate high-quality, single-contig genome assemblies for two PWB phytoplasma reference strains, enabling robust comparative genomics. Employing advanced comparative genomics, our objectives were to identify genomic features, their phylogenetic position, analyze differences and conserved genome regions, structural plasticity, characterize variation in effector gene repertoires, other pathogenesis-related genomic features, including mobile group II intron abundance and horizontal gene transfer events.

## Materials and Methods

### Sample collection, phytoplasma identification, and quantification

Samples of *Parthenium hysterophorus* plants exhibiting typical phytoplasma symptoms, including phyllody and witches' broom, were collected from areas surrounding soybean and other agricultural fields across multiple locations in western Maharashtra, India. Collected plant samples were cleaned immediately, and stored at -80°C until further processing. Phytoplasma infection was confirmed by nested PCR targeting the 16S rRNA gene (Kirdat et al., 2022), and the resulting amplicons were sequenced and assembled for identification. Closely related strains were determined through EzBioCloud database comparisons, and phytoplasma titers were quantified using TaqMan-based qPCR (Christensen et al., 2004).

### Enrichment of phytoplasma DNA and genome sequencing

Genomic DNA (~ 1 µg) from phytoplasma infected parthenium leaves (PR08, PR34) was quantified using a Qubit 4 Fluorometer (Thermo Fisher Scientific, USA) and quality checked by agarose gel electrophoresis and Nanodrop spectrophotometry. Prokaryotic DNA was enriched with the NEBNext Microbiome DNA Enrichment Kit (E2612L, New England BioLabs, USA) and amplified using the Ready To Go GenomiPhi V3 Kit (GE25 6601 96, Merck, Germany). Sequencing libraries were prepared with the NEBNext Ultra II DNA Library Prep kit, and paired end 150 bp reads were generated on an Illumina NovaSeq 6000 instrument. For long read sequencing, DNA was ligation prepared using the SQK LSK109 kit and run on an Oxford Nanopore MinION with R10.3 (Q20+) flow cells. In parallel, two alternative DNA processing workflows were used: (i) samples were treated with Long Fragment Buffer (LFB) from the ONT SQK‑LSK109 ligation sequencing kit prior to enrichment and sequenced on Illumina NovaSeq 6000, or (ii) genomic DNA was first purified using Genomic‑tips 20/G (QIAGEN) before enrichment and sequenced directly on the ONT MinION platform. Enrichment efficiency was evaluated by TaqMan qPCR targeting plant 18S and phytoplasma 16S rRNA genes (Christensen et al., 2004) using a StepOnePlus Real-Time PCR System (Applied Biosystems, USA); samples showing >10-fold reduction in host DNA with increased phytoplasma signal were selected for sequencing.

### Genome assembly and annotations

Illumina reads were quality-trimmed and filtered along with ONT reads using standard quality-control pipelines prior to hybrid assembly. Hybrid genome assemblies were generated with Unicycler (Wick et al., 2017) and scaffolded using MeDuSa (Bosi et al., 2015) with reference to related complete phytoplasma genomes available in GenBank. Assemblies were validated by read remapping using Bowtie2 (Langmead and Salzberg, 2012) and minimap2 (Li, 2018) and evaluated with QUAST (Gurevich et al., 2013). Final assemblies were polished using Pilon (Walker et al., 2014) for short-read correction and Medaka (Oxford Nanopore Technologies) for long-read consensus refinement. Assembly quality was further assessed using CheckM (Parks et al., 2015) to estimate genome completeness and contamination. To validate phytoplasma enrichment and remove host contamination, all raw reads were taxonomically classified using Kaiju (Menzel et al., 2016); only reads assigned to phytoplasmas were retained for hybrid assembly. Genome annotation was performed using Prokka (Seemann, 2014), PGAP (Tatusova et al., 2016) and Bakta (Schwengers et al., 2021), and species-level relatedness was assessed via OrthoANI (Yoon et al., 2017) and digital DNA-DNA hybridization using GGDC (Auch et al., 2010). Functional categorization was achieved through eggNOG and KEGG annotations, and putative virulence factors were identified through BLASTP queries against VFDB (Chen et al., 2005). To assess antioxidant systems, we screened Bakta-derived annotations across 252 complete phytoplasma genomes for open reading frames encoding superoxide dismutase (SOD) and thioredoxin domain–containing proteins. Domain presence was validated via InterProScan (v5.76-107) using default settings.

### Effector protein prediction and characterization

Coding sequences annotated by Bakta (Schwengers et al., 2021) were screened against a curated set of validated phytoplasma effectors; for example, SAP05, SAP11, and SAP54, TENGU (Gan et al., 2025; MacLean et al., 2014). Candidates meeting defined thresholds (coverage ≥ 30%, identity ≥ 30%, bit-score ≥ 50, E-value ≤ 1e-3) were retained after redundancy filtering. These effector-like proteins were analyzed for secretion signals using SignalP v6.0 (Teufel et al., 2022), transmembrane domains with TMHMM (Krogh et al., 2001), and nuclear localization signals using NLS Mapper (Kosugi et al., 2009). High-confidence effectors were structurally modeled with AlphaFold2 (Jumper et al., 2021), compared to reference effectors using Foldseek (van Kempen et al., 2024), and visualized in PyMOL, with model confidence assessed using AlphaFold2 pLDDT scores. Root-mean-square deviation (RMSD) values and aligned atom counts were calculated from the resulting structural superpositions. Evolutionary relationships were inferred via MUSCLE alignments (Edgar, 2004), and Neighbor-Joining phylogenies constructed in MEGA12 (Kumar et al., 2024) with 1,000 bootstrap replicates.

### Phylogenetic position of PR08 and PR34 and PCoA analysis

Phylogenetic placement of PR08 and PR34 was assessed using MEGA12 (Kumar et al., 2024) with Neighbor-Joining, Maximum-Likelihood, and Maximum-Parsimony analyses of 16S rRNA gene sequences. Whole genome based phylogeny was performed using core genes identified by BPGA (Chaudhari et al., 2016), and 89 conserved marker genes from UBCG (Na et al., 2018), with HMMER searches (Potter et al., 2018) for marker detection. To validate genome-based placement, additional trees were constructed using five informative marker genes: [replication initiation protein DnaD (*dnaD*), DegV family protein (*degV*), TIGR00282 family metallophosphoesterase, preprotein translocase SecY (*secY*), and RluA family pseudo-uridine synthase (*rluA*)] (Cho et al., 2020).

Comparative gene content was examined with OrthoMCL clustering (Fischer et al., 2011), yielding 476 shared gene clusters across selected phytoplasma genomes. These were converted into a Jaccard distance matrix using VEGAN in R (Dixon, 2003), and Principal Coordinates Analysis (PCoA) was performed with APE (Paradis and Schliep, 2019). The reference set included Sesame phyllody SS02 (JAHBAJ02; Ranebennur et al., 2022), Peanut Witches’ broom NTU2011 (NZ_AMWZ01; Chung et al., 2013), *Echinacea purpurea* witches’-broom phytoplasma NCHU2014 (CP040925; Tan et al., 2021), and ‘*Ca*. Phytoplasma citri’ WBDL (MWKN01). Highly fragmented and low-quality PWB group genomes were excluded.

### Characterization of group II intron encoded proteins

Group II intron-encoded proteins (IEPs) in PR34 and PR08 were first identified from genome annotations generated by Prokka (Seemann, 2014), Bakta (Schwengers et al., 2021), and PGAP (Tatusova et al., 2016). To improve accuracy, we compiled a curated reference set of 723 bacterial IEPs covering canonical classes (ML, CL, A–F), expanded subclasses (g2–g5), and newly described clades U1–U3 and G2I-L1–L4 (Candales et al., 2012; Miura et al., 2022). All available phytoplasma genomes were re-annotated using Bakta, and predicted proteins were screened with InterProScan to confirm hallmark domains, including reverse transcriptase (RT), maturase (Domain X), and DNA endonuclease. For comparative analysis, a non-redundant dataset of 52 representative genomes was constructed by excluding closely related ‘*Ca*. P. australasiaticum’ strains to avoid overrepresentation bias.

Candidate IEPs were identified through BLASTp searches against the curated reference set using stringent filters (≥ 30% coverage and identity, bit score ≥ 50, E-value ≤ 1e-3). Redundant matches were collapsed, resulting in 226 unique phytoplasma IEPs. These were classified according to the scheme of Zimmerly and Semper (2015) into mitochondrial-like (ML), chloroplast-like (CL), bacterial A–F, and extended clades U1–U3 and G2I-L1–L4 (Candales et al., 2012; Miura et al., 2022).

Representative sequences (n = 254) were aligned in Clustal Omega, and phylogenetic analysis was performed in MEGA12 (Kumar et al., 2024) using the Neighbor-Joining method with the JTT+G model and 1000 bootstrap replicates. Conserved RT motifs (blocks 0–7) and Domain X were visualized in Jalview. Genome-wide intron distributions were quantified using Python libraries (pandas, matplotlib, and seaborn) and mapped for PR08, PR34, and *Echinacea purpurea* witches’-broom phytoplasma NCHU2014 (Tan et al., 2021) using DNA Features Viewer and Proksee (Grant et al., 2023). Local neighborhoods of RT–Domain X pairs were examined to identify potential insertion hotspots.

## Results and Discussion

### Phytoplasma identification and selection

Partial 16S rRNA gene sequences from 27 *Parthenium hysterophorus* plants with phyllody symptoms confirmed infection by peanut witches’ broom (PWB; 16SrII) phytoplasmas. Twelve samples shared 99.02–100% identity with ‘*Ca*. P. asiaticum’, while 15 showed 99.28–100% identity with ‘*Ca*. P. australasiaticum’ (formerly ‘*Ca*. P. australasia’). Despite their phylogenetic distinction, both lineages produced indistinguishable symptoms across locations. All 16S rRNA gene sequences used for classification were derived from high-quality Sanger chromatograms, and no mixed base calls or ambiguous peaks were observed, indicating the absence of mixed infections in the surveyed samples. Two representative isolates were selected for complete genome sequencing: PR08 (LN879443), identical to ‘*Ca*. P. australasia’ strain Y10097 (Carica papaya), and PR34 (MZ724173), clustering with ‘*Ca*. P. citri’. These strains were later designated as reference material for ‘*Ca*. P. australasiaticum’ and ‘*Ca*. P. asiaticum’, respectively, in the formal genome-based species description by Jardim et al. (2023), which relied on ANI and dDDH thresholds. Although PR34 initially showed ~99.38% similarity to ‘*Ca*. P. citri’, comparative genomic analysis revealed it to be a distinct lineage within 16SrII-C. As the genome of strain Carica papaya (Y10097) remains unavailable, sequencing PR08 provided a relevant comparator to assess genome-level divergence within the 16SrII group and later assigned as reference strain for ‘*Ca*. P. australasiaticum’ (Jardim et al., 2023).

### Prokaryotic DNA enrichment and genome sequencing

**Fig. 1.**
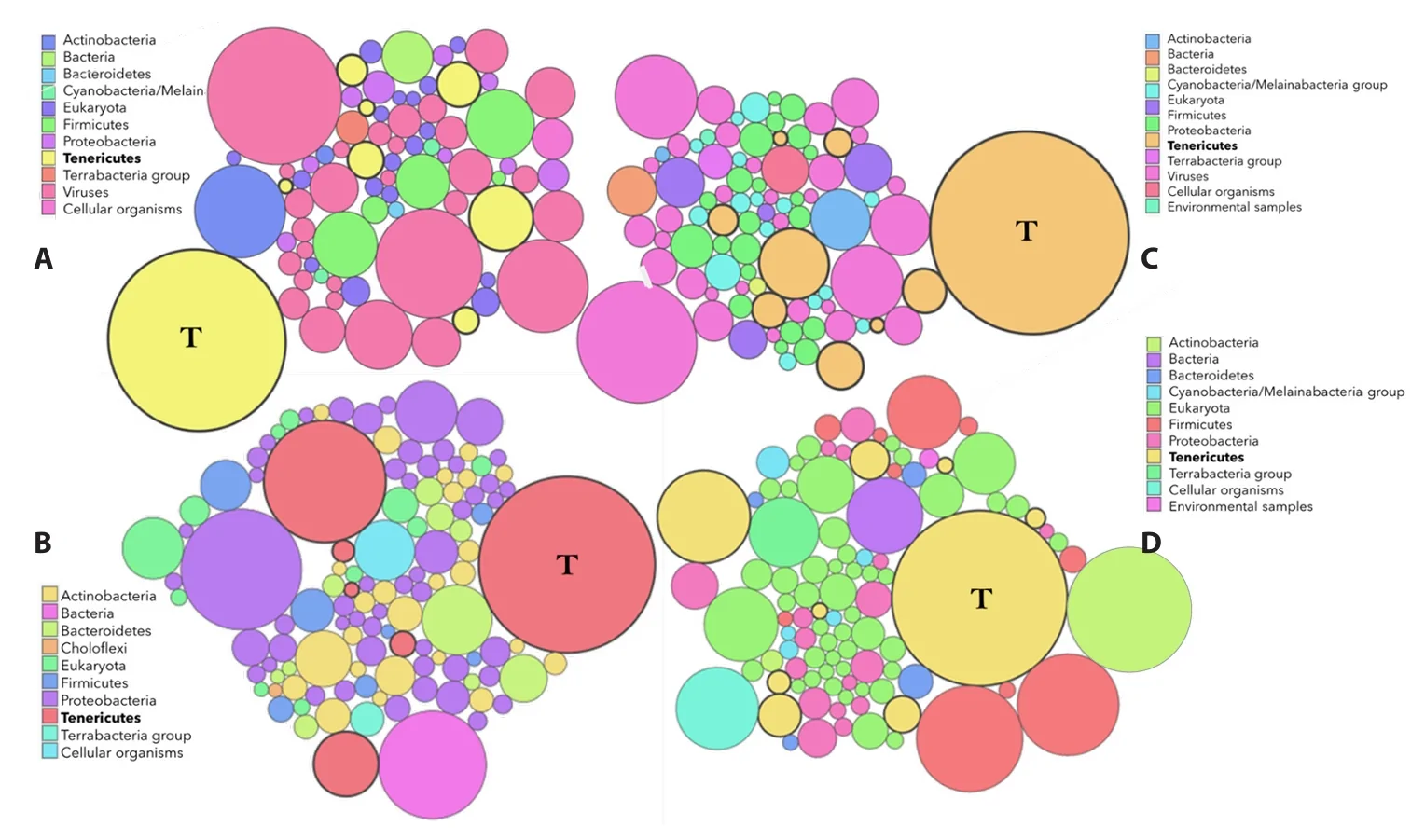
Kaiju-based taxonomic classification of enriched DNA from isolates PR08 and PR34. Bubble plots show the number and relative abundance of taxa based on raw Illumina reads (A, C) and Oxford Nanopore reads (B, D). Bubble size is scaled logarithmically to the number of reads assigned to each taxon. Prominent assignment to *Tenericutes* (T) indicates substantial phytoplasma DNA, while the reduced representation of ‘Eukaryota’ highlights effective host DNA depletion.

For PR34, Illumina sequencing of NEBNext-enriched and Illustra-amplified DNA produced 54 million reads but a fragmented assembly (134 contigs; GCA_015100165.1), reflecting amplification bias and host contamination. Taxonomic profiling of raw reads using Kaiju confirmed substantial phytoplasma enrichment with minimal plant or microbial contamination (Fig. 1). Only reads classified as phytoplasma were retained for hybrid assembly. Incorporation of Long Fragment Buffer (LFB) before enrichment improved contiguity (33 contigs; GCA_015100165.2). The most effective strategy combined Qiagen Genomic-tip 20/G purification, NEBNext enrichment, and Oxford Nanopore sequencing, yielding 601,709 ONT reads. Hybrid assembly with Illumina data (MeDuSa, IGV curation) produced a complete 614,574 bp circular chromosome (CP097206.1) with 5700X Illumina and 180X ONT coverage. The obtained assemblies were further polished with Pilon (Illumina correction) and Medaka (Nanopore consensus) to mitigate errors introduced by multiple displacement amplification (MDA), and long-read artifacts. Amplification artifacts from MDA were addressed by using multiple DNA replicates, and only consistently supported regions were retained after polishing.

For PR08, ONT-only sequencing of NEBNext-enriched DNA produced a circular genome (CP060385) but with annotation errors and low completeness (52.68% by CheckM). A hybrid strategy using Illumina reads from LFB-purified DNA (18.5 million) and 389,506 ONT reads improved assembly (five contigs; JAGXLX01). Final optimization with Qiagen Genomic-tip purification yielded 39.8 million Illumina reads, and hybrid assembly via Unicycler produced a complete 588,746 bp circular chromosome (CP097207) with 2758X Illumina and 73X ONT coverage.

The final assemblies of PR34 and PR08 showed uniform, high-depth Illumina and Nanopore read coverage across single circular chromosomes, with Kaiju-based read classification indicating negligible non-phytoplasma signal, supporting their interpretation as single, dominant phytoplasma genomes rather than composite assemblies. CheckM analysis indicated high completeness and negligible contamination for both assemblies (Table 1). These results show that phytoplasma genome quality is strongly influenced by DNA processing and sequencing strategy. Qiagen Genomic-tip purification with NEBNext enrichment improved host DNA removal for Illumina sequencing, while ONT reads were essential for spanning repeat-rich regions, particularly those encoding multiple reverse transcriptase proteins. An optimized hybrid approach combining high-depth Illumina with long ONT reads is recommended for generating accurate, complete phytoplasma genomes.

### Genome statistics

The genome of phytoplasma isolate PR34 (CP097206) consists of a single circular chromosome of 614,574 bp, with a G + C content of 24.65%. It encodes 474 protein-coding sequences (CDSs), two rRNA operons, 28 tRNA genes, and 18 pseudogenes. In comparison, isolate PR08 (CP097207), also associated with parthenium phyllody, harbors a compact circular genome of 588,746 bp, currently the smallest reported among 16SrII phytoplasmas with a slightly lower G + C content of 24.36%. This genome contains 468 CDSs, two rRNA operons, 27 tRNA genes, and 15 pseudogenes (Table 1). Consistent with advanced genome reduction seen in obligate phytoplasmas, both PR34 and PR08 exhibit hallmark features of reductive evolution, including small genome sizes, reduced coding densities, loss of intact PMUs, and retention of only core virulence and stress-adaptation genes (Table 1 and below). Functional categorization using the Clusters of Orthologous Groups (COG) database assigned 304 genes in PR34 (64.1%) and 352 in PR08 (75.2%) to 19 functional categories. In both genomes, the most represented category was translation, ribosomal structure, and biogenesis, comprising 104 genes in PR34 and 109 in PR08, followed by genes involved in transport and metabolism; 70 and 71 genes, respectively (data not shown).

PR34 exhibits the lowest coding density (70.61%) among currently sequenced 16SrII phytoplasma genomes, compared to PR08 (72.74%) and other peanut witches’-broom strains such as NCHU2014 (71.88%), NTU2011 (73.31%), SS02 (72.25%), and WBDL (73.76%) (Table 1). This supports a pattern of pronounced genome erosion in PR34, possibly reflecting advanced reductive evolution in a host-adapted niche (Bobay and Ochman, 2017). Interestingly, PR34 also encodes the highest number of tRNA genes reported in 16SrII phytoplasmas to date, possibly reflecting the selective retention of translational adaptability under conditions of genome minimization (Khomarbaghi et al., 2024). Orthologous cluster analysis using OrthoVenn2 revealed distinct differences in gene content across PWB phytoplasmas. Both the cluster distribution and presence–absence matrices indicated isolate-specific protein repertoires, offering quantitative insights into shared core functions and lineage-specific adaptations (Fig. S1). These differences likely represent evolutionary responses to divergent ecological niches and host plant associations.

### Phylogenetic analysis and OGRI-based taxonomic delineation

**Fig. 2.**
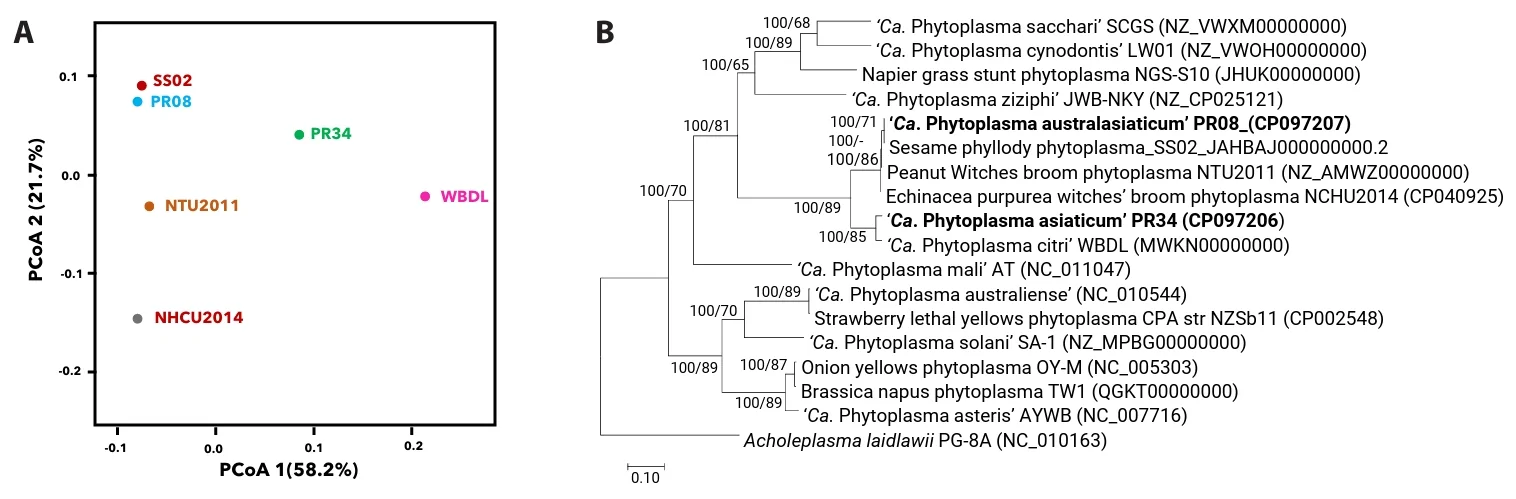
Genome-wide phylogenetic and gene content analyses of PWB group phytoplasmas. Principal Coordinate Analysis (PCoA) based on Jaccard distances derived from 476 OrthoMCL gene clusters among six 16SrII (PWB) phytoplasma genomes. PR34 (‘*Ca*. P. asiaticum’) forms a distinct cluster, indicating significant divergence in gene content compared to other PWB isolates. The first two principal coordinates, PC1 and PC2, explain 58.2% and 21.7% of the total variance, respectively (A); Pan-genome phylogeny of phytoplasmas based on orthologous protein-coding genes identified using BPGA and UBCG pipelines. The BPGA tree was constructed using MEGA12 (Neighbour-Joining, 1,000 bootstrap replicates) from 10,387 amino acid positions, while the UBCG tree used 86,628 positions across 89 marker genes predicted via Prodigal and HMMER. Bootstrap support from both methods is shown at nodes. *Acholeplasma laidlawii* PG-8A served as the outgroup (B).

Principal Coordinate Analysis (PCoA) based on pairwise comparisons of 476 genes of six phytoplasma genomes within the PWB (16SrII) group viz. ‘*Ca*. P. asiaticum’ PR34, ‘*Ca*. P. citri’ WBDL, ‘*Ca*. P. australasiaticum’ PR08, SS02, and NCHU2014, and NTU2011 (Peanut Witches’ Broom) which revealed five distinct clusters. Five distinct genomic clusters were observed among the analyzed PWB phytoplasma genomes, with PR34 forming an outlying group. Notably, Indian-origin isolates of ‘*Ca*. P. australasiaticum’, such as PR08 and SS02, clustered separately, suggesting regional genomic diversification within the lineage (Fig. 2A). PCoA, which reflects overall genomic dissimilarity, thus suggested substantial divergence of PR34 from other PWB phytoplasmas. This clustering pattern identified by PCoA was independently validated by phylogenetic analyses. Nearly full-length 16S rRNA gene-based phylogeny placed PR34 in a distinct clade, separate from ‘*Ca*. P. citri’ (WBDL) and ‘*Ca*. P. australasiaticum’ (PR08) (Fig. S2). Phylogenetic trees based on whole-genome metrics (Fig. 2B) and concatenated housekeeping genes (Fig. S3) support the distinction of PR34 and PR08 as separate subclades within the 16SrII group and indicate divergent evolutionary paths characterized by strain-specific mobile element profiles and gene content reduction.

Orthologous genome relatedness indices (OGRI) were computed to quantify the genomic distinctiveness of PR34. The average nucleotide identity (orthoANI) values between PR34 and ‘*Ca*. P. citri’ (WBDL) and ‘*Ca*. P. australasiaticum’ (NCHU2014) were 95.52% and 86.25%, respectively. Digital DNA-DNA hybridization (dDDH) values, calculated using formula 2 of the GGDC method, were 62.1% and 31.6%, respectively (Table 1). These values fall below the species delineation thresholds of 96% for ANI and 70% for dDDH (Meier-Kolthoff et al., 2013), supporting the recognition of PR34 as a genomically distinct lineage within the 16SrII group. This aligns with the genome-based delimitation previously proposed by Jardim et al. (2023), in which PR34 served as the reference strain for ‘*Ca*. P. asiaticum’.

### Genome features

The parthenium phyllody phytoplasma isolates PR08 (16SrII-D) and PR34 (16SrII-C) show distinct genomic traits compared to other PWB phytoplasmas. These include a diverse set of Sec-secreted effectors, absence of large PMUs, retention of uncommon genes such as *sodA* (superoxide dismutase) and truncated hlyB transporters, and an unusually high number of group II introns. The following sections describe these features and their implications for pathogenicity, host and vector interactions, and genome plasticity, with comparisons to related 16SrII strains (NTU2011, SS02, WBDL, NCHU2014).

### Presence of a wide array of secretory proteins

The genomes of PR08 and PR34 encode a broad repertoire of Sec-dependent effector proteins that are central to phytoplasma virulence. These small, secreted proteins are delivered via the Sec secretion system and reprogram host physiology, influencing plant development, immunity, and vector transmission (Bai et al., 2009; MacLean et al., 2014). Genome annotation with Bakta v1.5.0 identified the complete protein-coding complement of PR08 and PR34. Local BLASTp searches against a curated effector database yielded 19 high-confidence hits. After removing redundant entries, eight unique effector candidates were retained in PR08 and seven in PR34. Both strains encoded homologs of the canonical SAP11 effector: PR34 (UQV27401) and PR08 (UQV26586) shared ~41% identity with the Aster Yellows Witches’ Broom (AYWB) SAP11, supporting their classification as functional orthologs.

To refine predictions, candidate proteins were screened for secretion signals and structural hallmarks. SignalP v6.0 identified strong signal peptides in several proteins, including UQV26928.1 (PR08) and UQV27395.1 (PR34), both with probabilities > 0.85 and predicted cleavage sites between residues 23–28. TMHMM analysis confirmed the absence of transmembrane domains, strengthening their designation as soluble secretory proteins. Nuclear localization signals were predicted in multiple candidates; for example, the PR34 SAP11 homolog (UQV27401) had a strong NLS score of 7.9, while the PR08 SAP11 homolog (UQV26586) scored 5.6. Another high-confidence protein, SAP08 (UQV27393 in PR34), also carried a robust NLS, indicating nuclear targeting potential. In total, 11 of the 15 predicted effectors across both genomes satisfied all criteria for Sec secretion, lack of transmembrane domains, and nuclear localization, defining them as high-confidence effectors.

**Fig. 3.**
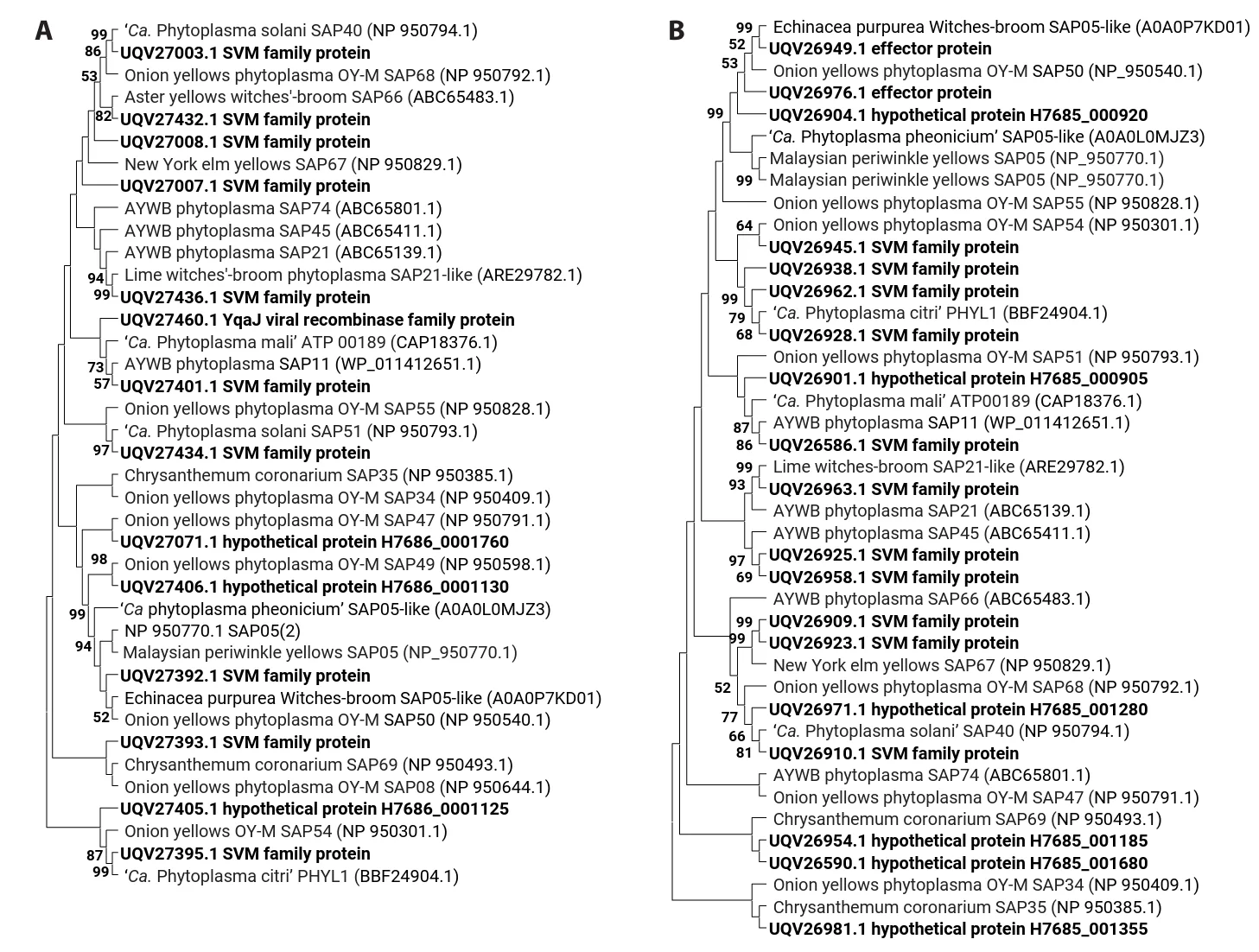
Phylogenetic relationships of predicted effector proteins from phytoplasma strains PR34 and PR08. Maximum likelihood tree of predicted secretory effector proteins from ‘*Ca*. P. asiaticum’ strain PR34 showing their clustering within canonical SAP families alongside known effectors from diverse phytoplasma lineages (A). Corresponding tree for ‘*Ca*. P. australasiaticum’ strain PR08, showing similar family-level associations and diversification of predicted SAP effectors (B). Predicted SAP-like proteins from PR34 and PR08 are highlighted in bold font. Each tree was independently constructed using strain-specific predicted SAP homologs identified via BLASTp searches and InterProScan domain annotations. Canonical reference SAP proteins from well-characterized phytoplasmas were selectively included based on top-scoring alignments to PR34 or PR08 sequences. Bootstrap support values above 50% are shown at nodes. Trees were constructed using maximum likelihood methods with representative sequences from previously characterized phytoplasma effectors.

Phylogenetic analyses provided further support for these assignments. Effector proteins from PR08 and PR34 clustered within canonical SAP families, while also revealing lineage-specific divergence (Fig. 3A and 3B). In PR34, the SAP11 homolog (UQV27401) grouped with shoot proliferation-associated variants from AYWB and ‘*Ca*. P. mali’, diverging from homologs in ‘*Ca*. P. citri’ (WBDL) and ‘*Ca*. P. australasiaticum’ (PR08), suggesting host-range adaptation. SAP54-like proteins UQV27395 and UQV27405 in PR34 clustered within the phyllody-associated clade but remained distinct from PHYL1 of ‘*Ca*. P. citri’ and OY-M. Similarly, UQV27406.1 and UQV27392 clustered with SAP05 proteins from ‘*Ca*. P. asteris’ and OY-M. In PR08, three homologs (UQV26949.1, UQV26976.1, UQV26904.1) aligned with SAP05-, SAP50-, and SAP55-like proteins from aster yellows phytoplasmas. Additional SAP11-related proteins (UQV26586.1, UQV26901.1) retained ~41% identity to AYWB and ‘*Ca*. P. mali’. SAP54-like candidates (UQV26945.1, UQV26938.1, UQV26962.1) grouped with the floral virescence-associated family. These clustering patterns indicate that while PR08 and PR34 maintain the canonical phytoplasma effector toolkit, their repertoires diverge in lineage-specific ways.

**Fig. 4.**
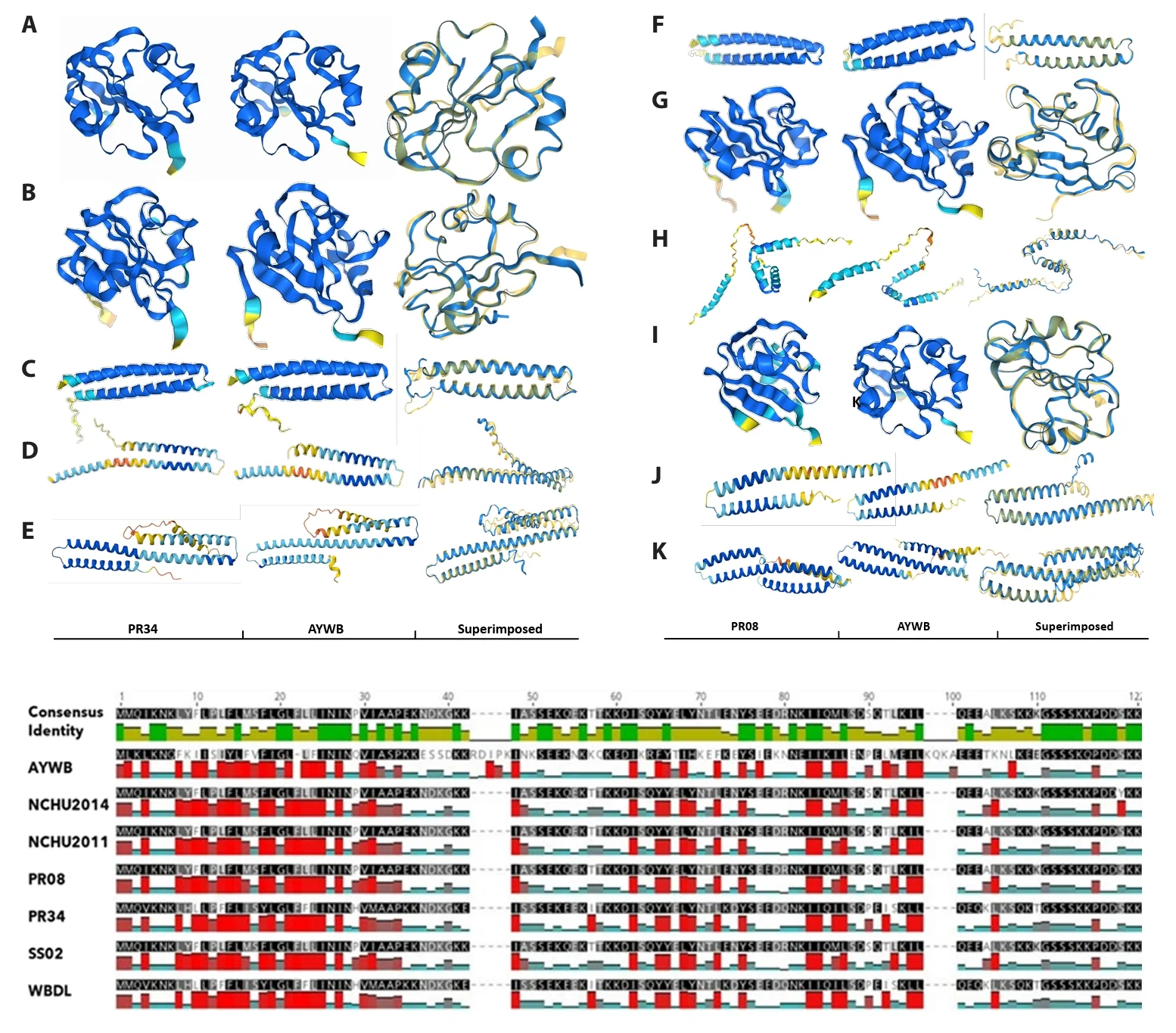
Structural and hydrophobicity-based comparison of SAP effectors from PR08 and PR34 with canonical AYWB phytoplasma homologs. Upper panel: AlphaFold2-predicted structures of SAP effector homologs from PR34 (A–E) and PR08 (F–K) were superimposed onto available reference crystal structures, including SAP05 (PDB: 8J48) and SAP54/PHYL1 (PDB: 6INR), using Foldseek. Structural superpositions show low RMSD values (0.40–0.71 Å), indicating strong conservation of the core α/β fold. Models are colored by pLDDT confidence, with high-confidence regions corresponding to conserved structural cores and lower-confidence regions largely restricted to signal peptides and terminal or loop regions. The PR34 proteins include SAP05 (UQV27392.1, A), SAP54/PHYL1 (UQV27395.1, B), SAP21-like (UQV27436.1, C), SAP19 (UQV27003.1, D), and SAP45-like (UQV27395.1, E); PR08 proteins include SAP54/PHYL1 (UQV26928.1, F), SAP05 (UQV26949.1, G), SAP11 (UQV26586.1, H), SAP05 variant (UQV26976.1, I), SAP21-like (UQV26963.1, J), and SAP45-like (UQV26958.1, K). Lower panel: Hydrophobicity plots of SAP11 homologs, illustrating conserved biophysical properties despite sequence divergence. Despite low sequence conservation, hydrophobicity profiles remain highly conserved, particularly across functionally relevant domains.

Structural analyses added an additional layer of validation. Comparative alignments and AlphaFold2-based structural predictions of SAP05, SAP11, SAP54 (PHYL1), SAP15, SAP19, SAP21-like, and SAP49 homologs from PR08 and PR34 revealed strong conservation of α/β folds across strains (Fig. 4). Foldseek-based comparisons confirmed near-identical structural cores, particularly across α-helices and β-sheets, with low RMSD values ranging from 0.40 to 0.71 Å across multiple effector–crystal structure pairings, supporting robust fold conservation despite modest sequence divergence. Deviations were largely confined to loop regions, which are structural elements often implicated in host protein–protein interactions. AlphaFold2 models of SAP11, SAP05, and SAP54 showed consistently high pLDDT scores across the conserved helical core (> 85–90), indicating high confidence in the predicted folds, while lower confidence was restricted to signal peptides and terminal regions. For example, PR34 and PR08 SAP05 and SAP54 homologs each showed low RMSD values (< 0.75 Å) when superimposed on their respective reference crystal structures, supporting strong fold conservation (Fig. 4). In SAP05, for example, subtle loop variability could influence binding to the plant ubiquitin–proteasome system, while the PR34 SAP11 homolog closely mirrored AYWB SAP11 in hydrophobicity profiles and overall fold, suggesting functional mimicry of TCP-binding activity (Fig. 4). These models therefore provide reliable structural context for effector conservation, although functional interactions remain to be experimentally validated.

Beyond canonical SAP proteins, both genomes also encode several hypothetical proteins (e.g., UQV26901.1, UQV26954.1, UQV26981.1 in PR08; UQV27405.1 in PR34) that cluster within effector families and carry Sec signals, supporting their designation as candidate effectors. A substantial subset belongs to the sequence-variable mosaic (SVM) family, comprising small, rapidly evolving proteins often linked to host adaptation (Carreón-Anguiano et al., 2023).

Together with conserved SAP11, SAP05, and SAP54 homologs, these findings suggest a potential dual virulence strategy: a stable core of effectors that target conserved host pathways; such as, SAP11 destabilizing TCP transcription factors (Pecher et al., 2019), SAP05 manipulating proteasomal degradation (Huang et al., 2021a), and SAP54 inducing floral virescence (MacLean et al., 2014) and a flexible set of lineage-specific effectors that enable adaptation to diverse plant hosts and vectors. While this framework is consistent with domain predictions and prior functional studies in other phytoplasmas, the precise roles of these effectors in PR34 and PR08 remain to be experimentally validated. This balance of structural conservation with adaptive diversification underscores how streamlined phytoplasma genomes maintain a robust yet versatile virulence repertoire.

### Antioxidant defense systems

Phytoplasmas, as obligate intracellular parasites of plants and insects, must navigate host-generated oxidative stress. In plants, pathogen perception triggers an oxidative burst characterized by rapid production of reactive oxygen species (ROS) such as superoxide (O₂^⁻^) and hydrogen peroxide (H₂O₂), which serve both antimicrobial and signaling roles (Dumanović et al., 2021). Despite their reduced genomes, phytoplasmas retain core antioxidant components, notably superoxide dismutase (SOD) and the thioredoxin (Trx) system, both implicated in oxidative stress management. In PR34 and PR08 genomes, we identified intact *sodA* genes (UQV27294 and UQV26792), encoding Mn/Fe-type SODs with conserved metal-binding motifs. Similar retention of *sodA* has been documented in ‘*Ca*. Phytoplasma asteris’ strains OY-M and AY-WB, ‘*Ca*. P. mali’, and others (Tran-Nguyen et al., 2008), reflecting a conserved requirement for superoxide detoxification.

To assess the broader conservation of these systems, we analyzed 252 phytoplasma genomes annotated using Bakta (Table S3). Among these, 234 genomes encoded at least one SOD-domain–containing protein, and 213 encoded at least one thioredoxin-domain–containing protein. Notably, all *sodA*-containing strains also retained at least one thioredoxin-like gene, suggesting functional linkage. For example, ‘*Ca*. Phytoplasma pruni’ isolate PR2021 (GCA_029746895.1) encoded five thioredoxin-domain proteins. Seven genomes (e.g., GCA_030587215.1, GCA_001189415.1, GCA_032886195.1, etc.) lacked *sodA* but retained thioredoxin, while one genome (GCA_012519065.1) lacked both SOD and canonical peroxidase-like proteins but encoded a thiol peroxidase. Biochemical characterization of *sodA* in OY phytoplasma confirmed Mn-SOD activity and its expression in both host types (Miura et al., 2012). The absence of sequence-variable mosaic (SVM) domains in PR34 and PR08 *sodA* suggests a cytoplasmic localization, consistent with a primary intracellular role. PR08, along with 135 other phytoplasma genomes, encodes a lipoyl-dependent peroxidase/OsmC-Ohr family protein (IPR003718, IPR036102, IPR015946) while all genomes lacked catalase genes. Additionally, PR34 also lacks peroxidase encoding genes.

Peroxidases and catalases are enzymes that typically detoxify the H_2_O_2_ generated by SOD activity. This absence raises the question of alternative mechanisms for H_2_O_2_ clearance. In contrast to some mycoplasmas that encode hydroperoxide reductases (Saikolappan et al., 2009), phytoplasmas appear to rely on a different strategy. Supporting this, PR34 and PR08 encode thioredoxin-like proteins (UQV27055 and UQV26564), components of the Trx/TrxR system, which has been implicated in peroxide detoxification and redox maintenance in *Mycoplasma pneumoniae* (Ben-Menachem et al., 1997). The co-occurrence of *sodA* and Trx system components suggests a complementary antioxidant network that compensates for the absence of classical peroxide-scavenging enzymes. Recent transcriptomic analyses have further highlighted the functional relevance of *sodA*. In ‘*Ca*. P. ziziphi’, *sodA* was strongly upregulated during early colonization stages, correlating with bacterial titers and proposed ROS suppression (Xue et al., 2023). Such observations support the idea that *sodA* plays a critical role in enabling phytoplasma persistence in both phloem and insect environments.

In addition to its enzymatic function, *sodA* may have evolved moonlighting roles, as reported in other bacteria. These include participation in immune evasion, redox signaling, and DNA protection (Wang et al., 2014). Although such functions remain unproven in phytoplasmas, the potential exists, particularly given their streamlined genomes and lack of redundancy in stress-response pathways. The consistent co-retention of *sodA* and thioredoxin-domain proteins across most phytoplasma genomes further supports the idea that *sodA* potentially operates as part of a minimal yet robust oxidative stress defense module. Its evolutionary persistence, despite the loss of accessory ROS-detox systems such as catalases and peroxidases, indicates both its essentiality for intracellular survival and its possible multifunctionality in redox adaptation.

### Presence of multiple and truncated hemolysin genes

In Gram-negative bacteria such as *Escherichia coli*, hemolysin secretion relies on a Type I Secretion System (T1SS) formed by *hlyB*, *hlyD*, and the outer membrane channel *tolC* genes, which export *hlyA* in a single step (Thomas et al., 2014). Phytoplasmas, however, are Mollicutes bounded only by a single plasma membrane and lacking an outer membrane and peptidoglycan (Razin and Hayflick, 2010). This architecture precludes the existence of a canonical T1SS, and accordingly, neither PR34 nor PR08 encodes HlyA, HlyC, HlyD, or TolC proteins.

In PR34 and PR08, multiple *hlyB*-like genes were identified; seven in PR34 and thirteen in PR08, but no homologs of *hlyA*, *hlyC*, *hlyD*, or *tolC* were detected. These HlyB-like proteins retain the conserved ABC transporter ATP-binding domain but lack full-length transmembrane segments or accompanying operon components. The presence of adjacent gene pairs (UQV27256-57 in PR34) suggests tandem duplication events. The absence of hemolysin III (HlyIII) or RTX-family pore-forming toxins further distinguishes PR34 and PR08 from certain other phytoplasmas like ‘*Ca*. P. australiense’ or JWB, where divergent hemolysin-like ORFs have been observed. Importantly, in the absence of key structural partners and secretion substrates, moonlighting roles for HlyB-like proteins are unlikely, as no known examples exist in bacteria where isolated ABC transporter subunits acquire unrelated biological functions.

Also retained in both genomes is SpoVG, a conserved regulatory protein implicated in coordinating cell division, stress responses, and virulence gene expression, annotated as a “septation protein,” in both PR34 and PR08 genomes (UQV27195.1 and UQV26692.1). In *Bacillus subtilis*, disruption of *spoVG* gene abolishes hemolytic activity (Pan et al., 2014), while in *S. aureus* and *L. monocytogenes*, SpoVG regulates toxin production, biofilm formation and other virulence factors (Shi et al., 2023; Xu et al., 2025). Although PR34 and PR08 lack functional hemolysins, *spoVG*’s retention suggests broader roles, possibly in replication, stress adaptation, or membrane regulation (Benthien et al., 2022; Burke and Portnoya, 2016; Huang et al., 2021b). Together, the multiple truncated *hlyB*-like genes and the conserved *spoVG* regulator reflect a legacy of functional systems shaped by genome erosion, with structural and genomic constraints making functional T1SS-mediated toxin secretion implausible.

### Absence of canonical PMUs and scattered mobile elements

Phytoplasma genomes are typically shaped by PMUs, replicative composite elements that act as key hubs for genome plasticity, horizontal gene transfer (HGT), and effector mobility (Ku et al., 2013; Oshima et al., 2004). Recent comparative analyses highlight that PMUs are the *principal engines* of phytoplasma genome dynamism and effector diversification, with more than 60% of characterized effectors embedded within PMU regions that together occupy less than 12% of the genome (Gan et al., 2025). These 10–20 kb multicopy clusters typically include replication (*ssb*, *dnaB*, and *dnaG*), recombination (*himA*), and metabolic (*tmk*) genes flanked by insertion sequences such as *tra5*, and can occur in both chromosomal and extrachromosomal forms. Through frequent duplication, recombination, and horizontal transfer, PMUs provide an unparalleled platform for effector innovation and host-adaptation events (Bai et al., 2009; Chung et al., 2013; Gan et al., 2025).

In sharp contrast, PR34 and PR08 lack any intact PMU assemblies. No contiguous gene blocks encoding the full PMU complement were detected. Instead, only isolated remnants containing pseudogenes for IS3-family transposases (*tra5*), degenerate sigma-70 factors, and single copies of *ssb*, *hflBMM*, *tmk*, *dnaB*, *dnaG*, and *himA* are scattered across their compact genomes. These fragments retain recognizable domains but appear in variable orientations without synteny, indicating extensive degradation. This differs markedly from the ~20 kb PMU1 of AYWB phytoplasma (NC_007716) and the partially intact PMUs still present in ‘*Ca*. P. mali’ (Kube et al., 2008). The disintegration of these mobile modules suggests minimal potential for further PMU-mediated HGT or rapid structural rearrangement.

From an evolutionary standpoint, PR34 and PR08 likely represent a terminal stage of PMU decay and immobilization. Intact PMUs likely once contributed to gene mobility and effector acquisition but have since been eroded through deletion and pseudogenization, leaving behind static, dispersed relics (Chung et al., 2013). Notably, some phyllody-inducing effectors such as SAP54-typically PMU-linked in other phytoplasmas, are still retained, implying strong purifying selection for essential virulence determinants despite the collapse of surrounding mobile DNA. This PMU-deficient architecture appears to diverge sharply from the PMU-centric model of phytoplasma genome evolution (Gan et al., 2025), suggesting that intron-associated or residual transposase-mediated rearrangements could now serve as the primary, though limited, routes for genome restructuring. Such genomes may exemplify a minimal yet functional evolutionary state in which long-term stability and selective retention of vital effectors compensate for the loss of canonical mobile-unit dynamics.

### Presence of self-catalytic, high-copy group II introns

**Fig. 5.**
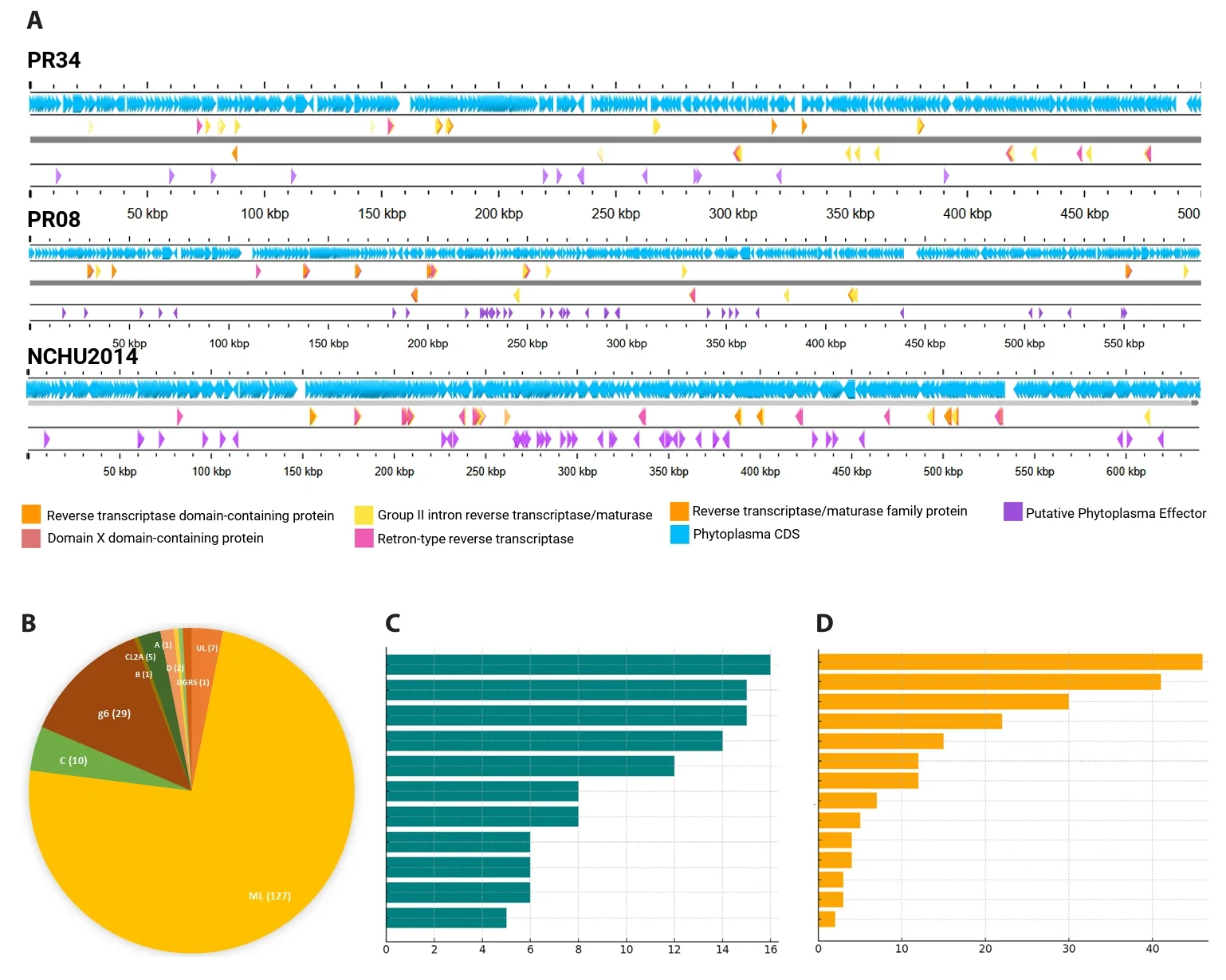
Proliferation and diversity of Group II introns in phytoplasma genomes. Genome maps of PR08, PR34, and NCHU2014 showing distribution of group II intron-encoded reverse transcriptases (RTs) (colored arrows) and associated maturase domains, including intact, split, and pseudo-ORF configurations (A). Classification of 226 phytoplasma intron ORFs reveals dominance of the mitochondrial-like (ML) class, with other classes occurring infrequently (B). Comparative abundance of intron-encoded RTs across top 15 phytoplasma genomes, highlighting the unusually high copy numbers in PWB group members (C) and Top 15 non-phytoplasma prokaryotic genomes containing similar intron classes, suggesting possible horizontal gene transfer sources (D). See Table S3 for full list of genome sequences employed for this analysis.

Despite extensive degradation of PMUs in PR34 and PR08, both genomes harbor an unusually high abundance of another class of mobile elements: group II introns. These ribozymes, typically encoding a reverse transcriptase (RT)–maturase protein, are self-propagating elements known to influence genome plasticity proteins (Lambowitz and Zimmerly, 2011; Zimmerly and Semper, 2015). Their prominence in these phytoplasmas indicates that even in the absence of intact PMUs, mobile DNA continues to shape genome architecture. Automated annotation with Bakta predicted 28 intron-encoded ORFs in PR08 and 52 in PR34, far exceeding the corresponding NCBI PGAP calls of 4 and 14, respectively (Fig. 5A, Tables S1–S3). This discrepancy likely reflects the divergence and partial decay of many intron ORFs, which are missed by conservative annotation pipelines (Candales et al., 2012).

Domain analysis confirmed that most Bakta-predicted ORFs contained hallmark RT blocks 0–7 and, in many cases, the downstream maturase domain (Domain X), consistent with canonical group II intron-encoded proteins (IEPs). InterPro signatures confirmed the presence of both RT (IPR013750) and RNA-binding/maturase domains (IPR013749) in all 52 PR34 ORFs and the majority in PR08 (Fig. S4). A striking feature of PR08 was the occurrence of split ORFs, where RT and Domain X were encoded by adjacent but distinct genes within a few hundred bp (Fig. S4A). Upstream intergenic regions (~500–600 bp) of these loci contained sequence motifs consistent with intron RNA domains (I–III), suggesting partial intron decay (Michel and Ferat 1995). In contrast, PR34 encoded mostly intact single-ORF IEPs harboring both RT and Domain X (Fig. S4B). PR34 also carried a subset of pseudo-RTs disrupted by frameshifts or premature stops, marking ongoing inactivation.

Classification of 226 unique phytoplasma intron ORFs against reference IEPs revealed a striking dominance of the mitochondrial-like (ML) class of group II introns. Of these, 167 (~74%) were ML-type, while other classes viz. D, g6, C, and A, were each represented by fewer than 30 instances. Chloroplast-like (CL2A/CL2B), diversity-generating retroelement (DGR)-associated RTs, and minor F subtypes (g2–g5) appeared only sporadically, typically with 1–2 occurrences per genome (Fig. 5B, Tables S1–S3). Only one PR34 locus was assigned to RNA class IIB/IEP class B. This pattern mirrors the findings of Miura et al. (2022), who showed that ML-class introns predominate across phytoplasma genomes. The enrichment of ML introns, which encode RT, maturase, and often endonuclease domains, suggests that they remain functional and mobile despite the streamlined nature of phytoplasma chromosomes.

A survey of available phytoplasma genomes confirmed that prolific intron content is characteristic of the peanut witches’ broom (PWB; 16SrII) lineage. Every examined 16SrII genome carried multiple introns, whereas most other phytoplasmas contained none or at most one. A few non-PWB strains, including ‘*Ca*. P. solani’ 231/09, Mulberry dwarf, and ‘*Ca*. P. australiense’ SLY, contained more than one, with SLY carrying eight ltrA-like ORFs, five of them fragmented (Luo et al., 2022; Music et al., 2019). Thus, the intron proliferation observed in PR34 and PR08 is not a general property of phytoplasmas but a lineage-specific expansion within PWB strains (Fig. 5C, Table S3).

Multiple lines of evidence support horizontal gene transfer (HGT) as the origin of phytoplasma group II introns. First, BLASTp comparisons show that phytoplasma intron-encoded RTs are more similar to proteins from distant bacteria like *Novosphingobium aromaticivorans*, *Bacillus cereus*, and *Alteromonas macleodii* than to those from Mollicutes (Tourasse and Kolstø, 2008). Several ML-class introns in PR08 and PR34 matched *N. aromaticivorans* intron proteins with ~33% identity over 90% of the length. Second, these intron regions exhibit elevated GC content (~36–37%) compared to the AT-rich phytoplasma genome (~21–28%), a hallmark of recent HGT from higher-GC donors (Ochman et al., 2000). Third, phylogenetic analysis of 254 RT sequences revealed a distinct phytoplasma clade on long branches, diverging from other bacterial introns and absent from *Acholeplasma*, which lacks group II introns altogether. Introns from PR08, PR34, and related PWB strains clustered tightly, suggesting a shared origin. Together, these findings indicate that phytoplasma introns are recent acquisitions from foreign sources, not vertically inherited.

In PR34 and PR08, intron-related sequences account for > 4% of the total genome (Darby et al., 2007; this study), a substantial fraction in chromosomes of only ~0.6 Mb. Group II introns are generally regarded as selfish elements that self-splice and retrohome into new sites, expanding independently of host benefit (Lambowitz and Belfort, 2015). We infer that one or more active ML introns entered PWB phytoplasmas via phage- or plasmid-mediated transfer and subsequently underwent rapid intragenomic amplification. Although intron mobility is often facilitated by PMUs or phage-like elements in other phytoplasmas (Wei et al., 2008), PR34 and PR08 lack intact PMUs. Their intron proliferation therefore occurred despite PMU decay, likely through direct retrohoming or recombination within repeat-rich SVM regions. The intron repertoires of PR34 and PR08 illustrate different evolutionary states. PR08 encodes many split RT/X ORFs and fragmented RNA domains, indicative of ongoing degeneration. By contrast, PR34 contains more intact full-length ORFs, though several pseudo-RTs suggest inactivation is also underway. Both genomes show intron enrichment within repeat-rich SVM loci (Table S3), where homology could promote recombination and copy-number variation.

While group II introns are primarily parasitic, their insertions can influence local regulation or create recombinogenic hotspots. In bacteria, introns have been implicated in altering gene expression and facilitating genome plasticity under stress or host shifts (Zimmerly and Semper, 2015). In PR34 and PR08, the high intron load may provide a reservoir of variability that offsets the constraints of extreme genome reduction. However, such proliferation could also impose replication costs or disrupt coding regions, representing a balance between adaptability and genomic burden.

Together, these findings highlight group II introns as a potential contributor of genome architecture in PWB phytoplasmas. In PR34 and PR08, more than 80 intron-encoded ORFs, predominantly of the ML class, have proliferated to occupy a significant fraction of the genome, contrasting sharply with the absence or rarity of introns in other phytoplasma lineages. Their phylogenetic distinctness, elevated G + C content, and similarity to introns from unrelated bacteria support a plausible scenario of recent horizontal acquisition followed by intragenomic expansion. Importantly, this expansion occurred despite the collapse of canonical PMUs, raising the possibility that group II introns may function as the dominant mobile elements contributing to genome variability in these lineages. The presence of both intact and decaying introns is consistent with a dynamic equilibrium between mobility and loss, shaping genome evolution in minimal bacteria. Collectively, PR34 and PR08 illustrate how streamlined pathogens may reconcile reductive evolution with bursts of mobile element activity, leaving a genomic landscape where introns, rather than PMUs, appear to represent the prevailing source of variability (Figs. 5A–5C, and S4; Tables S1–S3).

## Conclusion

The complete genomes of PR34 (‘*Ca*. P. asiaticum’) and PR08 (‘*Ca*. P. australasiaticum’) illustrate a notable feature of phytoplasma evolution: extreme reductive streamlining paired with selective retention and even expansion of mobile genetic elements. The collapse of canonical PMUs contrasts with the unprecedented proliferation of group II introns, likely acquired through recent horizontal transfer, which appear to substantively contribute to genome variability and highlight alternative routes to plasticity in minimal bacteria. Despite severe gene loss, both genomes conserve a virulence toolkit centered on canonical effectors SAP05, SAP11, and SAP54. Their structural conservation is consistent with functional stability, while lineage-specific divergence and additional candidate effectors point to adaptive flexibility of phytoplasmas in manipulating plant hosts and insect vectors. The retention of *sodA* within a pared-down antioxidant network likewise reflects selective pressure for oxidative stress management despite the loss of classical peroxide-detoxifying enzymes. Other features, including multiple truncated *hlyB*-like loci and the conserved regulator SpoVG, represent vestiges of more complex ancestral systems. While no longer functional in secretion, these remnants provide clues to past capacities and potential regulatory roles. Together, these findings position PR34 and PR08 as models of advanced reductive evolution, balancing genome stability with innovation, and highlight effector complements and intron dynamics as promising targets for future functional investigation.

## Figures and Tables

**Table 1.** Comparative genomic features of phytoplasma strains within the Peanut Witches’ Broom (PWB; 16SrII) group. This table summarizes the genomic characteristics of six PWB group strains: ‘*Ca*. P. asiaticum’ (PR34), ‘*Ca*. P. australasiaticum’ (PR08, SS02, NTU2011, and NCHU2014), and ‘*Ca*. P. citri’ (WBDL). Key parameters include genome size, number of contigs, protein-coding genes, rRNA and tRNA gene counts, coding density, and G + C content. Genomic relatedness between PR34 and other isolates was assessed using OrthoANI, digital DNA-DNA hybridization (dDDH), and the percentage of shared genome segments.

Isolate ID	Accession number	No. of contigs	Genome size (bp)	Proteins	rRNA	tRNA	Coding density	% G + C	ANI	dDDH	Genome shared (%)
PR34	CP097206	1	614,574	474	6	28	70.61	24.65	100	100	100^*^
PR08	CP097207	1	588,746	468	6	27	72.74	24.36	86.38	30.5	77.45
NCHU2014	CP040925	1	639,808	471	6	24	71.88	24.54	86.25	31.6	77.45
NTU2011	AMWZ01	14	566,694	448	6	27	73.31	24.37	85.81	30.8	75.00
SS02	JAHBAJ01	60	553,228	449	3	17	72.25	23.68	85.64	30.6	68.62
WBDL	MWKN01	98	474,669	385	0	19	73.76	23.9	95.52	62.1	58.33

* The raw alignment value of 99.01% arises from minor technical artifacts associated with low-confidence alignments in the circularization overlap region of the PR34 assembly. These discrepancies reflect known limitations in short-read polishing and fragment mapping near contig ends. For interpretive consistency, we present the theoretical genome-shared value of 100% for self-comparison.

## Data Availability

The GenBank/EMBL/DDBJ accession numbers for the reference 16S rRNA gene sequences of phytoplasma isolates PR34 and PR08 are MZ724173 and LN879443. The accession number of complete genomes are CP097206 and CP097207. The versions described in this manuscript are CP097206.1 and CP097207.1. Other partial 16S rRNA gene sequences were submitted under accession numbers HG792252, LN878981, 82; LN879437 to 43; LT558766 to 69; LT558783, 84, 89; MG748740 to 45; MT555411, 12; MT940950 to 69; MZ724173 and 74. A compressed archive of Bakta-annotated GenBank files for all phytoplasma genomes analyzed (PR34, PR08, and 52 other complete genomes); The full Bakta annotation tables, capturing all CDSs, including intron-encoded reverse transcriptase/maturase ORFs; Custom scripts used to classify group II intron–associated loci are publicly available in a repository via Zenodo at https://doi.org/10.5281/zenodo.17857958.

## Supplementary Information

Table S1. Classified intron-encoded proteins identified in ‘*Ca*. P. australasiaticum’ strain PR08. Bakta annotation predicted 28 intron-encoded ORFs in PR08, including intact, split, and degenerate loci containing reverse transcriptase or maturase domains. Following BLASTp-based classification and redundancy filtering against a curated intron reference database, 15 non-redundant, high-confidence intron loci were retained and are listed here.

Table S2. Classified intron-encoded proteins identified in ‘*Ca*. P. asiaticum’ strain PR34. Bakta annotation predicted 52 intron-encoded ORFs in PR34, encompassing intact, split, and partially degenerate loci. After BLASTp-based classification and redundancy collapse against a curated intron reference database, 14 non-redundant, high-confidence intron loci were retained and are presented in this table.

Table S3. Source data used for preparation of Fig. 5 (panels B–D). This table contains the background and quantitative data used to generate Fig. 5B, 5C, and 5D in the manuscript, including classification, abundance, and comparative metrics of group II intron–encoded reverse transcriptases across selected phytoplasma and non-phytoplasma genomes. The data were used directly for plotting and statistical summaries shown in Fig. 5B–5D. No figures are presented within this table.

Fig. S1. Orthologous gene cluster comparison among PWB phytoplasma genomes using OrthoVenn2 (Xu et al., 2019). Genomes included: ‘*Ca*. P. citri’ WBDL (MWKN01), *Echinacea purpurea* witches’-broom phytoplasma NCHU2014 (CP040925), Peanut witches’-broom NTU2011 (NZ_AMWZ01), Parthenium phyllody phytoplasma PR08 (CP097207), ‘*Ca*. P. asiaticum’ PR34 (CP097206), and Sesame phyllody phytoplasma SS02 (JAHBAJ01). The occurrence matrix displays the top 20 shared orthologous groups, with species composition shown on the left. Cluster counts denote the number of shared ortholog groups, and protein counts indicate the number of proteins within those clusters, highlighting intergenomic variation in functional gene content.

Fig. S2. Phylogenetic position of ‘*Ca*. Phytoplasma asiaticum’ sp. nov. isolate PR34 inferred from analysis of reference 16S rRNA gene sequences of published provisional species of ‘*Ca*. Phytoplasma’. The Neighbour-Joining (NJ), Maximum-Likelihood (ML), and Maximum-Parsimony (MP) methods were employed, utilizing the Tajima-Nei, JTT, and Subtree-Pruning-Regrafting (SPR) models, respectively, in MEGA 12. The topologies of the trees were evaluated by bootstrap analysis based on 1,000 replicates. Bootstrap values are shown at nodes as NJ/ML/MP methods, separated by slashes; dashes indicate values < 50. There were a total of 1285 positions in the final dataset. The 16S rRNA sequence of *Acholeplasma laidlawii* PG-8A (M23932) was used as an outgroup. The bar indicates the number of nucleotide substitutions per site.

Fig. S3. Phylogenetic tree of concatenated full-length amino acid sequences of five individual housekeeping genes obtained from selected phytoplasma genomes. The tree involves five amino acid sequences of Replication initiation protein DnaD (*dnaD*), DegV family protein (*degV*), TIGR00282 family metallophosphoesterase, Preprotein translocase SecY (*secY*), and RluA family pseudo uridine synthase (*rluA*) genes totalling to 1132 positions in the final dataset. The evolutionary history was inferred by using the Maximum Likelihood method based on the Le_Gascuel_2008 model in MEGA 12. Figures at nodes of the branches indicate the percentage of replicate trees obtained from ML method. Bootstrap analysis was carried out using 1000 replicates. The respective sequences of *Acholeplasma laidlawii* PG-8A (NC 010163) were used as an outgroup. Bar indicates the number of substitutions per site.

Fig. S4. Domain architecture of Bakta-predicted group II intron-encoded proteins in PR08 (A) and PR34 (B). In PR08 (A), several reverse transcriptase (RT) ORFs exhibit split configurations where RT and maturase (Domain X) domains are encoded by consecutive but separate genes, with some RTs lacking adjacent Domain X. Upstream intergenic regions (~500–600 bp) may represent partial intron RNA remnants. In PR34 (B), all identified intron ORFs are intact, harboring both RT and Domain X within a single ORF, although a few pseudo-RT ORF sequences show disrupted motifs.

## References

[b1-jm-2511004] Auch AF, Klenk HP, Göker M (2010). Standard operating procedure for calculating genome-to-genome distances based on high-scoring segment pairs. Stand Genomic Sci.

[b2-jm-2511004] Bai X, Correa VR, Toruño TY, Ammar ED, Kamoun S (2009). AY-WB phytoplasma secretes a protein that targets plant cell nuclei. Mol Plant-Microbe Interact.

[b3-jm-2511004] Ben-Menachem G, Himmelreich R, Herrmann R, Aharonowitz Y, Rottem S (1997). The thioredoxin reductase system of mycoplasmas. Microbiology.

[b4-jm-2511004] Benthien H, Fresenborg B, Pätzold L, Elhawy MI, Huc-Brandt S (2022). The transcription factor SpoVG is of major importance for biofilm formation of *Staphylococcus epidermidis* under in vitro conditions, but dispensable for in vivo biofilm formation. Int J Mol Sci.

[b5-jm-2511004] Bobay LM, Ochman H (2017). The evolution of bacterial genome architecture. Front Genet.

[b6-jm-2511004] Bosi E, Donati B, Galardini M, Brunetti S, Sagot MF (2015). MeDuSa: a multi-draft-based scaffolder. Bioinformatics.

[b7-jm-2511004] Burke TP, Portnoya DA (2016). SpoVG is a conserved RNA-binding protein that regulates *Listeria monocytogenes* lysozyme resistance, virulence, and swarming motility. mBio.

[b8-jm-2511004] Candales MA, Duong A, Hood KS, Li T, Neufeld RAE (2012). Database for bacterial group II introns. Nucleic Acids Res.

[b9-jm-2511004] Carreón-Anguiano KG, Vila-Luna SE, Sáenz-Carbonell L, Canto-Canché B (2023). Novel insights into phytoplasma effectors. Horticulture.

[b10-jm-2511004] Chaudhari NM, Gupta VK, Dutta C (2016). BPGA-an ultra-fast pan-genome analysis pipeline. Sci Rep.

[b11-jm-2511004] Chen L, Yang J, Yu J, Yao Z, Sun L (2005). VFDB: a reference database for bacterial virulence factors. Nucleic Acids Res.

[b12-jm-2511004] Cho ST, Kung HJ, Huang W, Hogenhout SA, Kuo CH (2020). Species boundaries and molecular markers for the classification of 16SrI phytoplasmas inferred by genome analysis. Front Microbiol.

[b13-jm-2511004] Christensen NM, Nicolaisen M, Hansen M, Schulz A (2004). Distribution of phytoplasmas in infected plants as revealed by real-time PCR and bioimaging. Mol Plant-Microbe Interact.

[b14-jm-2511004] Chung WC, Chen LL, Lo WS, Lin CP, Kuo CH (2013). Comparative analysis of the peanut witches’-broom phytoplasma genome reveals horizontal transfer of potential mobile units and effectors. PLoS One.

[b15-jm-2511004] Darby AC, Cho NH, Fuxelius HH, Westberg J, Andersson SGE (2007). Intracellular pathogens go extreme: genome evolution in the Rickettsiales. Trends Genet.

[b16-jm-2511004] Dixon P (2003). VEGAN, a package of R functions for community ecology. J Veg Sci.

[b18-jm-2511004] Dumanović J, Nepovimova E, Natić M, Kuča K, Jaćević V (2021). The significance of reactive oxygen species and antioxidant defense system in plants: a concise overview. Front Plant Sci.

[b19-jm-2511004] Edgar RC (2004). MUSCLE: multiple sequence alignment with high accuracy and high throughput. Nucleic Acids Res.

[b20-jm-2511004] Fischer S, Brunk BP, Chen F, Gao X, Harb OS (2011). Using OrthoMCL to assign proteins to OrthoMCL-DB groups or to cluster proteomes into new ortholog groups. Curr Protoc Bioinformatics.

[b21-jm-2511004] Gan P, Yu X, Zou J, Li C, Yao Z (2025). Evolution and functional adaptation of phytoplasma effectors: a potential mobile unit-driven perspective. Plant Cell Environ.

[b22-jm-2511004] Grant JR, Enns E, Marinier E, Mandal A, Herman EK (2023). Proksee: in-depth characterization and visualization of bacterial genomes. Nucleic Acids Res.

[b23-jm-2511004] Gurevich A, Saveliev V, Vyahhi N, Tesler G (2013). QUAST: quality assessment tool for genome assemblies. Bioinformatics.

[b24-jm-2511004] Huang W, MacLean AM, Sugio A, Maqbool A, Busscher M (2021a). Parasitic modulation of host development by ubiquitin-independent protein degradation. Cell.

[b25-jm-2511004] Huang Q, Zhang Z, Liu Q, Liu F, Liu Y (2021b). SpoVG is an important regulator of sporulation and affects biofilm formation by regulating Spo0A transcription in *Bacillus cereus* 0–9. BMC Microbiol.

[b26-jm-2511004] Jardim BR, Tran-Nguyen LTT, Gambley C, Al-Sadi AM, Al-Subhi AM (2023). The observation of taxonomic boundaries for the 16SrII and 16SrXXV phytoplasmas using genome-based delimitation. Int J Syst Evol Microbiol.

[b27-jm-2511004] Jumper J, Evans R, Pritzel A, Green T, Figurnov M (2021). Highly accurate protein structure prediction with AlphaFold. Nature.

[b28-jm-2511004] Khomarbaghi Z, Ngan WY, Ayan GB, Lim S, Dechow-Seligmann G (2024). Large-scale duplication events underpin population-level flexibility in tRNA gene copy number in *Pseudomonas fluorescens* SBW25. Nucleic Acids Res.

[b29-jm-2511004] Kirdat K, Tiwarekar B, Sathe S, Yadav A (2023). From sequences to species: charting the phytoplasma classification and taxonomy in the era of taxogenomics. Front Microbiol.

[b30-jm-2511004] Kirdat K, Tiwarekar B, Swetha P, Padma S, Thorat V (2022). Nested real-time PCR assessment of vertical transmission of sandalwood spike phytoplasma (‘*Ca*. Phytoplasma asteris’). Biology (Basel).

[b31-jm-2511004] Kirdat K, Tiwarekar B, Thorat V, Sathe S, Shouche Y (2021). ‘*Candidatus* Phytoplasma sacchari’, a novel taxon - associated with sugarcane grassy shoot (SCGS) disease. Int J Syst Evol Microbiol.

[b32-jm-2511004] Kosugi S, Hasebe M, Matsumura N, Takashima H, Miyamoto-Sato E (2009). Six classes of nuclear localization signals specific to different binding grooves of importinα. J Biol Chem.

[b33-jm-2511004] Krogh A, Larsson B, Von Heijne G, Sonnhammer ELL (2001). Predicting transmembrane protein topology with a hidden Markov model: application to complete genomes. J Mol Biol.

[b34-jm-2511004] Ku C, Lo WS, Kuo CH (2013). Horizontal transfer of potential mobile units in phytoplasmas. Mob Genet Elements.

[b35-jm-2511004] Kube M, Schneider B, Kuhl H, Dandekar T, Heitmann K (2008). The linear chromosome of the plant-pathogenic mycoplasma ‘*Candidatus* Phytoplasma mali’. BMC Genomics.

[b36-jm-2511004] Kumar S, Stecher G, Suleski M, Sanderford M, Sharma S (2024). MEGA12: molecular evolutionary genetic analysis version 12 for adaptive and green computing. Mol Biol Evol.

[b37-jm-2511004] Lambowitz A, Belfort M (2015). Mobile bacterial group II introns at the crux of eukaryotic evolution. Microbiol Spectr.

[b38-jm-2511004] Lambowitz AM, Zimmerly S (2011). Group II introns: mobile ribozymes that invade DNA. Cold Spring Harb Perspect Biol.

[b39-jm-2511004] Langmead B, Salzberg SL (2012). Fast gapped-read alignment with Bowtie 2. Nat Methods.

[b40-jm-2511004] Li H (2018). Minimap2: pairwise alignment for nucleotide sequences. Bioinformatics.

[b41-jm-2511004] Luo L, Zhang X, Meng F, Wang Y, Zhou Y (2022). Draft genome sequence resources of mulberry dwarf phytoplasma strain MDGZ-01 associated with mulberry yellow dwarf (MYD) diseases. Plant Dis.

[b42-jm-2511004] MacLean AM, Orlovskis Z, Kowitwanich K, Zdziarska AM, Angenent GC (2014). Phytoplasma effector SAP54 hijacks plant reproduction by degrading MADS-box proteins and promotes insect colonization in a RAD23-dependent manner. PLoS Biol.

[b43-jm-2511004] Meier-Kolthoff JP, Auch AF, Klenk HP, Göker M (2013). Genome sequence-based species delimitation with confidence intervals and improved distance functions. BMC Bioinformatics.

[b44-jm-2511004] Menzel P, Ng KL, Krogh A (2016). Fast and sensitive taxonomic classification for metagenomics with Kaiju. Nat Commun.

[b45-jm-2511004] Michel F, Ferat JL (1995). Structure and activities of group II introns. Annu Rev Biochem.

[b46-jm-2511004] Miura MC, Nagata S, Tamaki S, Tomita M, Kanai A (2022). Distinct expansion of group II introns during evolution of prokaryotes and possible factors involved in its regulation. Front Microbiol.

[b47-jm-2511004] Miura C, Sugawara K, Neriya Y, Minato N, Keima T (2012). Functional characterization and gene expression profiling of superoxide dismutase from plant pathogenic phytoplasma. Gene.

[b48-jm-2511004] Music MS, Samarzija I, Hogenhout SA, Haryono M, Cho ST (2019). The genome of ‘*Candidatus* Phytoplasma solani’ strain SA-1 is highly dynamic and prone to adopting foreign sequences. Syst Appl Microbiol.

[b49-jm-2511004] Na SI, Kim YO, Yoon SH, Ha S, Baek I (2018). UBCG: up-to-date bacterial core gene set and pipeline for phylogenomic tree reconstruction. J Microbiol.

[b50-jm-2511004] Ochman H, Lawrence JG, Grolsman EA (2000). Lateral gene transfer and the nature of bacterial innovation. Nature.

[b51-jm-2511004] Oshima K, Kakizawa S, Nishigawa H, Jung HY, Wei W (2004). Reductive evolution suggested from the complete genome sequence of a plant-pathogenic phytoplasma. Nat Genet.

[b52-jm-2511004] Oshima K, Maejima K, Isobe Y, Endo A, Namba S (2023). Molecular mechanisms of plant manipulation by secreting effectors of phytoplasmas. Physiol Mol Plant Pathol.

[b53-jm-2511004] Pan X, Chen X, Su X, Feng Y, Tao Y (2014). Involvement of SpoVG in hemolysis caused by *Bacillus subtilis*. Biochem Biophys Res Commun.

[b54-jm-2511004] Paradis E, Schliep K (2019). Ape 5.0: an environment for modern phylogenetics and evolutionary analyses in R. Bioinformatics.

[b55-jm-2511004] Parks D, Imelfort M, Skennerton C, Hugenholtz P, Tyson G (2015). CheckM: assessing the quality of microbial genomes recovered from isolates, single cells, and metagenomes. Genome Res.

[b56-jm-2511004] Pecher P, Moro G, Canale MC, Capdevielle S, Singh A (2019). Phytoplasma SAP11 effector destabilization of TCP transcription factors differentially impact development and defense of Arabidopsis versus maize. PLoS Pathog.

[b57-jm-2511004] Potter SC, Luciani A, Eddy SR, Park Y, Lopez R (2018). HMMER web server: 2018 update. Nucleic Acids Res.

[b58-jm-2511004] Ranebennur H, Kirdat K, Tiwarekar B, Rawat K, Chalam VC (2022). Draft genome sequence of ‘*Candidatus* Phytoplasma australasia’ strain SS02 associated with sesame phyllody disease. 3 Biotech.

[b59-jm-2511004] Razin S, Hayflick L (2010). Highlights of mycoplasma research: an historical perspective. Biologicals.

[b60-jm-2511004] Saikolappan S, Sasindran SJ, Yu HD, Baseman JB, Dhandayuthapani S (2009). The *Mycoplasma genitalium* MG-454 gene product resists killing by organic hydroperoxides. J Bacteriol.

[b61-jm-2511004] Schwengers O, Jelonek L, Dieckmann MA, Beyvers S, Blom J (2021). Bakta: rapid and standardized annotation of bacterial genomes via alignment-free sequence identification. Microb Genomics.

[b62-jm-2511004] Seemann T (2014). Prokka: rapid prokaryotic genome annotation. Bioinformatics.

[b63-jm-2511004] Shi C, Zheng L, Lu Z, Zhang X, Bie X (2023). The global regulator SpoVG regulates *Listeria monocytogenes* biofilm formation. Microb Pathog.

[b64-jm-2511004] Tan CM, Lin YC, Li JR, Chien YY, Wang CJ (2021). Accelerating complete phytoplasma genome assembly by immunoprecipitation-based enrichment and MinION-based DNA sequencing for comparative analyses. Front Microbiol.

[b65-jm-2511004] Tatusova T, Dicuccio M, Badretdin A, Chetvernin V, Nawrocki EP (2016). NCBI prokaryotic genome annotation pipeline. Nucleic Acids Res.

[b66-jm-2511004] Teufel F, Almagro Armenteros JJ, Johansen AR, Gíslason MH, Pihl SI (2022). SignalP 6.0 predicts all five types of signal peptides using protein language models. Nat Biotechnol.

[b67-jm-2511004] Thomas S, Holland IB, Schmitt L (2014). The type 1 secretion pathway—the hemolysin system and beyond. Biochim Biophys Acta Mol Cell Res.

[b68-jm-2511004] Thorat V, Bhale U, Sawant V, More V, Jadhav P (2016). Alternative weed hosts harbor 16SrII group phytoplasmas associated with little leaf and witches’ broom diseases of various crops in India. Phytopathogenic Mollicutes.

[b69-jm-2511004] Tourasse NJ, Kolstø AB (2008). Survey of group I and group II introns in 29 sequenced genomes of the *Bacillus cereus* group: insights into their spread and evolution. Nucleic Acids Res.

[b70-jm-2511004] Tran-Nguyen LTT, Kube M, Schneider B, Reinhardt R, Gibb KS (2008). Comparative genome analysis of ‘*Candidatus* Phytoplasma australiense’ (subgroup *tuf*-Australia I; *rp*-A) and ‘*Ca*. Phytoplasma asteris’ strains OY-M and AY-WB. J Bacteriol.

[b71-jm-2511004] van Kempen M, Kim SS, Tumescheit C, Mirdita M, Lee J (2024). Fast and accurate protein structure search with Foldseek. Nat Biotechnol.

[b72-jm-2511004] Walker BJ, Abeel T, Shea T, Priest M, Abouelliel A (2014). Pilon: an integrated tool for comprehensive microbial variant detection and genome assembly improvement. PLoS One.

[b73-jm-2511004] Wang G, Xia Y, Cui J, Gu Z, Song Y (2014). The roles of moonlighting proteins in bacteria. Curr Issues Mol Biol.

[b74-jm-2511004] Wei W, Davis RE, Jomantiene R, Zhao Y (2008). Ancient, recurrent phage attacks and recombination shaped dynamic sequence-variable mosaics at the root of phytoplasma genome evolution. Proc Natl Acad Sci USA.

[b75-jm-2511004] White DT, Blackall LL, Scott PT, Walsh KB (1998). Phylogenetic positions of phytoplasmas associated with dieback, yellow crinkle and mosaic diseases of papaya, and their proposed inclusion in ‘*Candidatus* Phytoplasma australiense’ and a new taxon, ‘*Candidatus* Phytoplasma australasia’. Int J Syst Bacteriol.

[b76-jm-2511004] Wick RR, Judd LM, Gorrie CL, Holt KE (2017). Unicycler: resolving bacterial genome assemblies from short and long sequencing reads. PLoS Comput Biol.

[b77-jm-2511004] Xu L, Zhang X, Wang W, Shen J, Ma K (2025). The global regulator SpoVG is involved in biofilm formation and stress response in foodborne *Staphylococcus aureus*. Int J Food Microbiol.

[b78-jm-2511004] Xue C, Zhang Y, Li H, Liu Z, Gao W (2023). The genome of ‘*Candidatus Phytoplasma* ziziphi’ provides insights into their biological characteristics. BMC Plant Biol.

[b79-jm-2511004] Yadav A, Thorat V, Bhale U, Shouche Y (2015). Association of 16SrII-C and 16SrII-D subgroup phytoplasma strains with witches’ broom disease of *Parthenium hysterophorus* and insect vector *Orosius albicinctus* in India. Australas Plant Dis Notes.

[b80-jm-2511004] Yoon S, Ha S, Lim J, Kwon S, Chun J (2017). A large-scale evaluation of algorithms to calculate average nucleotide identity. Antonie Van Leeuwenhoek.

[b81-jm-2511004] Zimmerly S, Semper C (2015). Evolution of group II introns. Mob DNA.

[b82-jm-2511004] Zreik L, Carle P, Bové JM, Garnier M (1995). Characterization of the mycoplasma-like organism associated with witches’ broom disease of lime and proposition of a *Candidatus* taxon for the organism, ‘*Candidatus* Phytoplasma aurantifolia’. Int J Syst Bacteriol.

