# Supplementary material for: Genomic landscape reveals the dominance of self-catalytic, high-copy group II introns in PMU-deficient complete genomes of PWB phytoplasmas
Source: J Microbiol. 2026 Mar 19;64(4):e2511004. doi: 10.71150/jm.2511004 (PMC13577128; doi:10.71150/jm.2511004)
Supplement: Supplementary file 1 [file jm-2511004-Supplementary-Fig-S1.pdf]

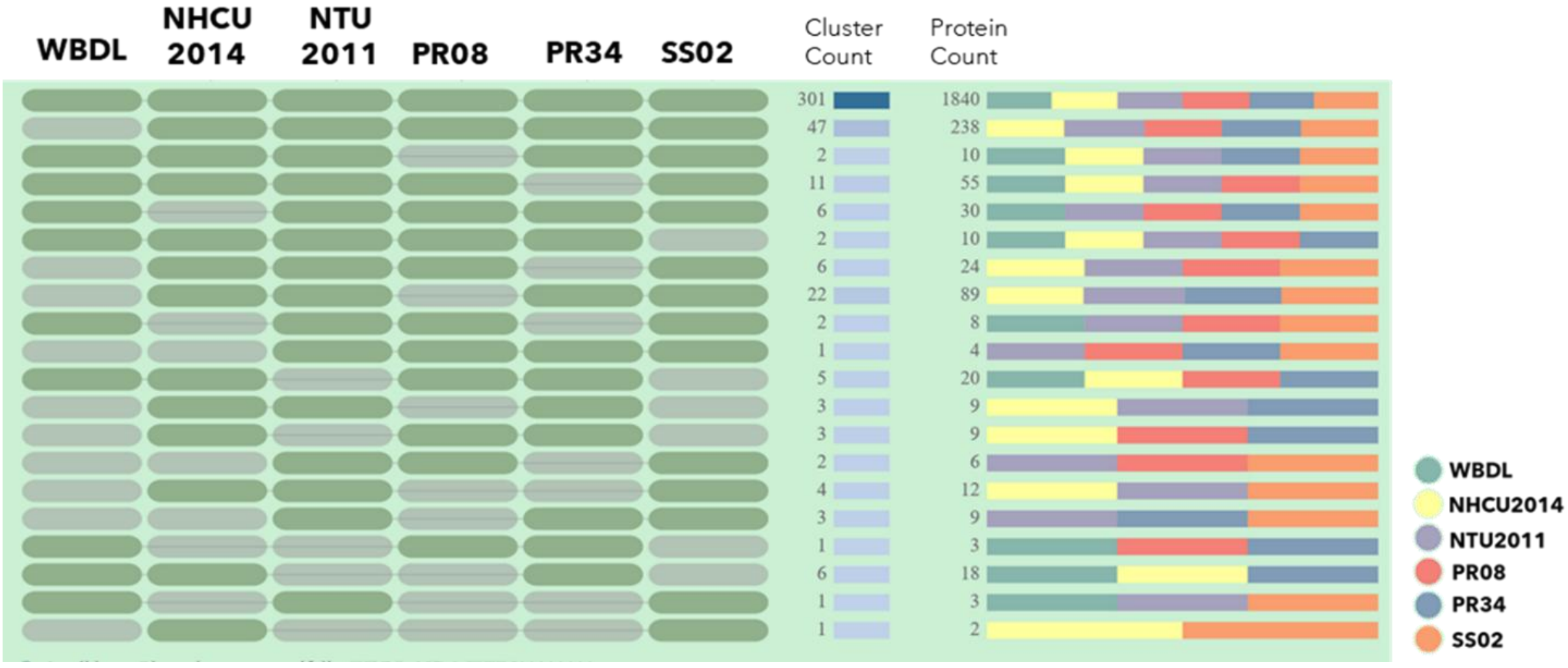

Fig. S1. Orthologous gene cluster comparison among PWB phytoplasma genomes using OrthoVenn2 (Xu et al., 2019). Genomes included: '*Ca*. P. citri' WBDL (MWKN01), *Echinacea purpurea* witches'-broom phytoplasma NCHU2014 (CP040925), Peanut witches'-broom NTU2011 (NZ_AMWZ01), Parthenium phyllody phytoplasma PR08 (CP097207), '*Ca*. P. asiaticum' PR34 (CP097206), and Sesame phyllody phytoplasma SS02 (JAHBAJ01). The occurrence matrix displays the top 20 shared orthologous groups, with species composition shown on the left. Cluster counts denote the number of shared ortholog groups, and protein counts indicate the number of proteins within those clusters, highlighting intergenomic variation in functional gene content.
