# Supplementary material for: Genomic landscape reveals the dominance of self-catalytic, high-copy group II introns in PMU-deficient complete genomes of PWB phytoplasmas
Source: J Microbiol. 2026 Mar 19;64(4):e2511004. doi: 10.71150/jm.2511004 (PMC13577128; doi:10.71150/jm.2511004)
Supplement: Supplementary file 2 [file jm-2511004-Supplementary-Fig-S2.pdf]

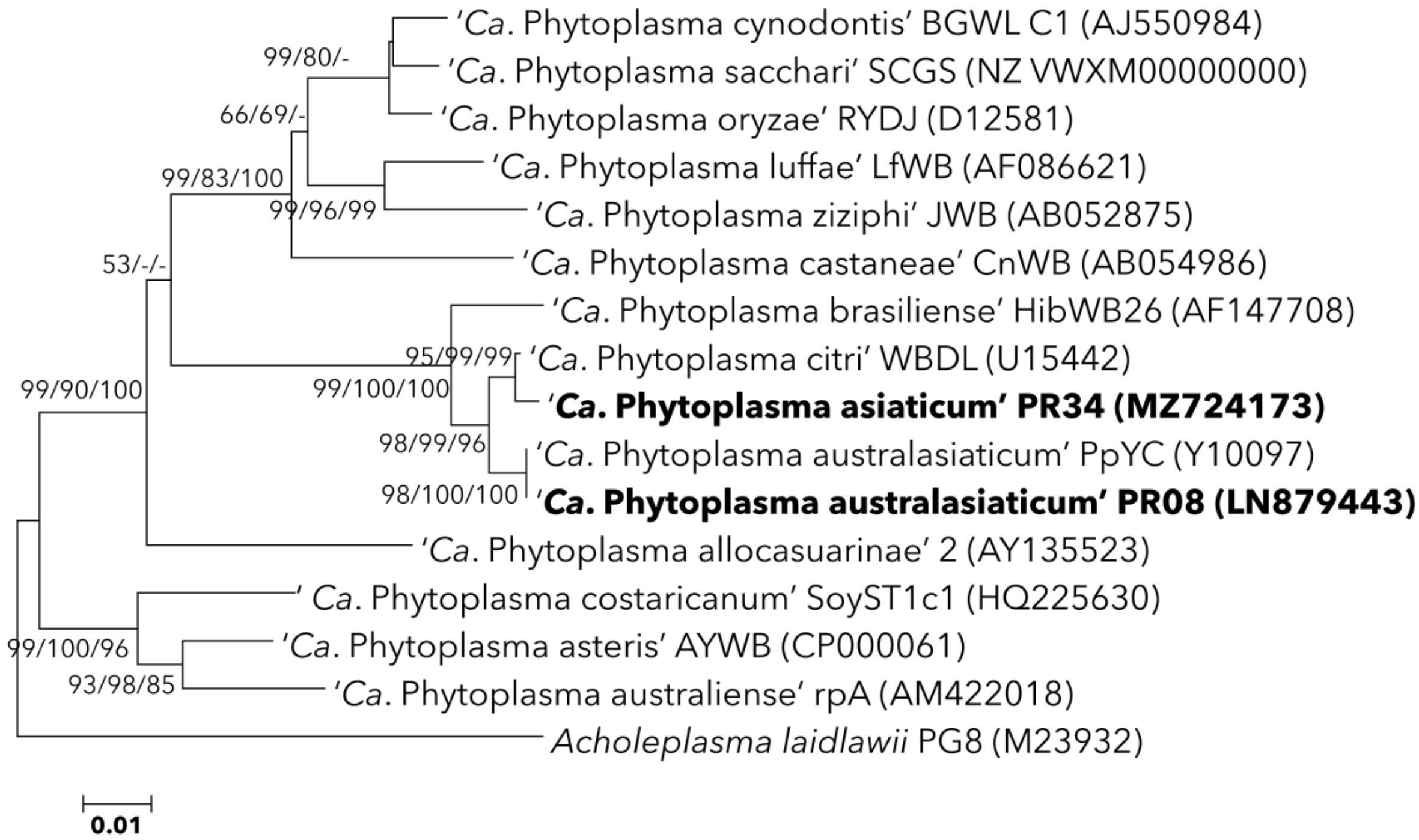

Fig. S2. Phylogenetic position of '*Ca.* Phytoplasma asiaticum' sp. nov. isolate PR34 inferred from analysis of reference 16S rRNA gene sequences of published provisional species of '*Ca.* Phytoplasma'. The Neighbour-Joining (NJ), Maximum-Likelihood (ML), and Maximum-Parsimony (MP) methods were employed, utilizing the Tajima-Nei, JTT, and Subtree-Pruning-Regrafting (SPR) models, respectively, in MEGA 12. The topologies of the trees were evaluated by bootstrap analysis based on 1,000 replicates. Bootstrap values are shown at nodes as NJ/ML/MP methods, separated by slashes; dashes indicate values < 50. There were a total of 1285 positions in the final dataset. The 16S rRNA sequence of *Acholeplasma laidlawii* PG-8A (M23932) was used as an outgroup. The bar indicates the number of nucleotide substitutions per site.
