# Supplementary material for: Genomic landscape reveals the dominance of self-catalytic, high-copy group II introns in PMU-deficient complete genomes of PWB phytoplasmas
Source: J Microbiol. 2026 Mar 19;64(4):e2511004. doi: 10.71150/jm.2511004 (PMC13577128; doi:10.71150/jm.2511004)
Supplement: Supplementary file 3 [file jm-2511004-Supplementary-Fig-S3.pdf]

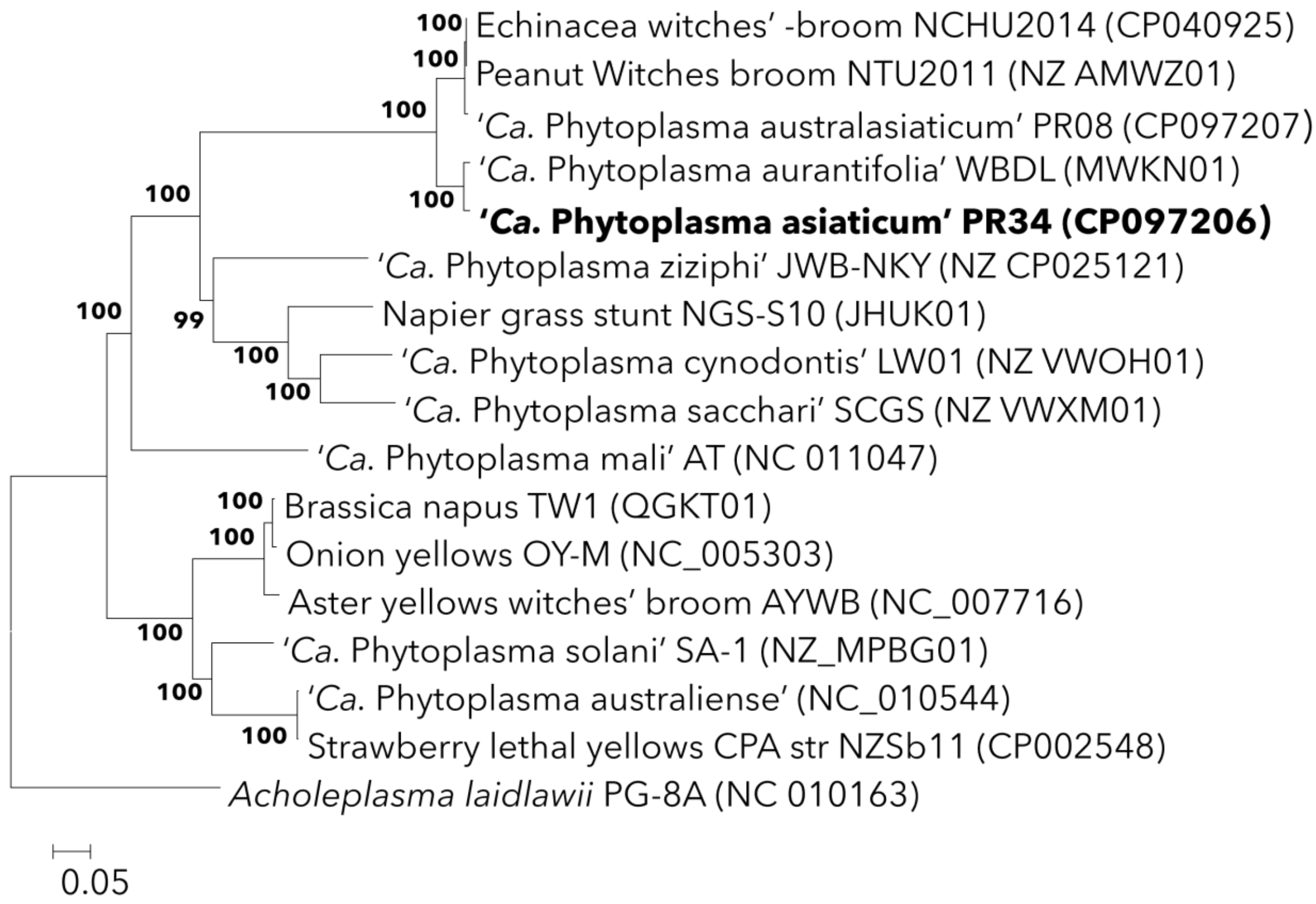

Fig. S3. Phylogenetic tree of concatenated full-length amino acid sequences of five individual housekeeping genes obtained from selected phytoplasma genomes. The tree involves five amino acid sequences of Replication initiation protein DnaD (*dnaD*), DegV family protein (*degV*), TIGR00282 family metallophosphoesterase, Preprotein translocase SecY (*secY*), and RluA family pseudo uridine synthase (*rluA*) genes totalling to 1132 positions in the final dataset. The evolutionary history was inferred by using the Maximum Likelihood method based on the Le_Gascuel_2008 model in MEGA 12. Figures at nodes of the branches indicate the percentage of replicate trees obtained from ML method. Bootstrap analysis was carried out using 1000 replicates. The respective sequences of *Acholeplasma laidlawii* PG-8A (NC 010163) were used as an outgroup. Bar indicates the number of substitutions per site.
