# Supplementary material for: Genomic landscape reveals the dominance of self-catalytic, high-copy group II introns in PMU-deficient complete genomes of PWB phytoplasmas
Source: J Microbiol. 2026 Mar 19;64(4):e2511004. doi: 10.71150/jm.2511004 (PMC13577128; doi:10.71150/jm.2511004)
Supplement: Supplementary file 4 [file jm-2511004-Supplementary-Fig-S4.pdf]

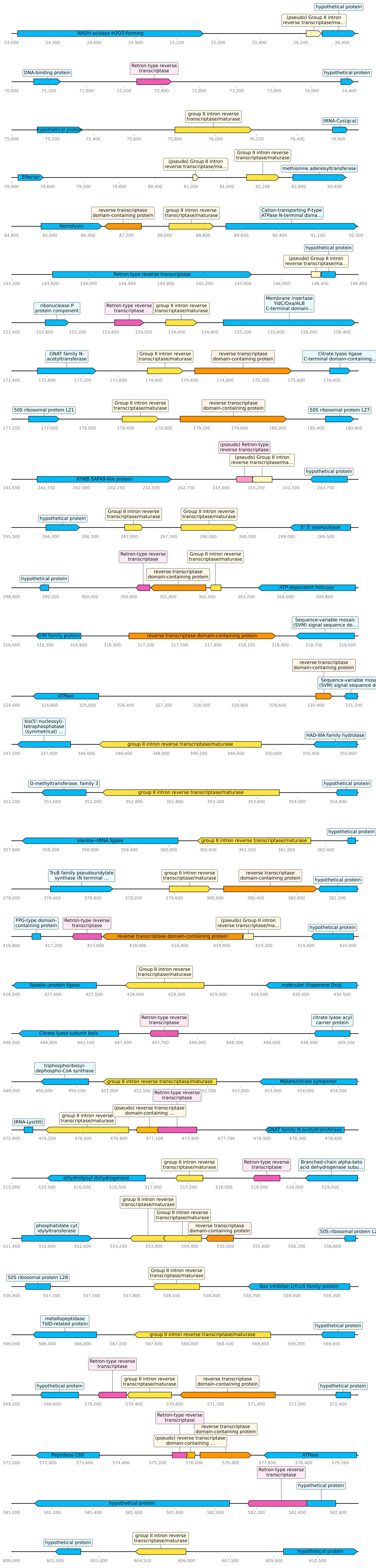
hypothetical protein
(pseudo) Group II intron reverse transcriptase/ma...
NADH oxidase H2O2-forming
24,000 24,300 24,600 24,900 25,200 25,500 25,800 26,100 26,400
DNA-binding protein
Retron-type reverse transcriptase
hypothetical protein
70,800 71,200 71,600 72,000 72,400 72,800 73,200 73,600 74,000 74,400
group II intron reverse transcriptase/maturase
hypothetical protein
tRNA-Cys(gca)
75,000 75,200 75,400 75,600 75,800 76,000 76,200 76,400 76,600
Group II intron reverse transcriptase/maturase
(pseudo) Group II intron reverse transcriptase/ma...
methionine adenosyltransferase
Effector
78,000 78,600 79,200 79,800 80,400 81,000 81,600 82,200 82,800 83,400
reverse transcriptase domain-containing protein
group II intron reverse transcriptase/maturase
Cation-transporting P-type ATPase N-terminal doma...
Hemolysin
84,800 85,600 86,400 87,200 88,000 88,800 89,600 90,400 91,200 92,000
hypothetical protein
(pseudo) Group II intron reverse transcriptase/ma...
Retron-type reverse transcriptase
143,200 143,600 144,000 144,400 144,800 145,200 145,600 146,000 146,400 146,800
Membrane insertase YidC/Oxa/ALB C-terminal domain...
ribonuclease P protein component
Retron-type reverse transcriptase
group II intron reverse transcriptase/maturase
152,400 152,800 153,200 153,600 154,000 154,400 154,800 155,200 155,600 156,000 156,400
GNAT family N-acetyltransferase
Group II intron reverse transcriptase/maturase
reverse transcriptase domain-containing protein
Citrate lyase ligase C-terminal domain-containing...
172,400 172,800 173,200 173,600 174,000 174,400 174,800 175,200 175,600 176,000
Group II intron reverse transcriptase/maturase
reverse transcriptase domain-containing protein
50S ribosomal protein L21
50S ribosomal protein L27
177,200 177,600 178,000 178,400 178,800 179,200 179,600 180,000 180,400 180,800
(pseudo) Retron-type reverse transcriptase
(pseudo) Group II intron reverse transcriptase/ma...
hypothetical protein
AYWB SAP49-like protein
241,500 241,750 242,000 242,250 242,500 242,750 243,000 243,250 243,500 243,750
Group II intron reverse transcriptase/maturase
Group II intron reverse transcriptase/maturase
hypothetical protein
5'-3' exonuclease
265,500 266,000 266,500 267,000 267,500 268,000 268,500 269,000 269,500
Retron-type reverse transcriptase
Group II intron reverse transcriptase/maturase
reverse transcriptase domain-containing protein
hypothetical protein
ATP-dependent helicase
298,400 299,200 300,000 300,800 301,600 302,400 303,200 304,000 304,800
Sequence-variable mosaic (SVM) signal sequence do...
SVM family protein
reverse transcriptase domain-containing protein
316,000 316,300 316,600 316,900 317,200 317,500 317,800 318,100 318,400 318,700 319,000
reverse transcriptase domain-containing protein
Sequence-variable mosaic (SVM) signal sequence do...
ATPase
324,000 324,800 325,600 326,400 327,200 328,000 328,800 329,600 330,400 331,200
bis(5'-nucleosyl)-tetraphosphatase (symmetrical) ...
HAD-IIIA family hydrolase
group II intron reverse transcriptase/maturase
347,200 347,600 348,000 348,400 348,800 349,200 349,600 350,000 350,400 350,800
O-methyltransferase, family 3
hypothetical protein
group II intron reverse transcriptase/maturase
351,200 351,600 352,000 352,400 352,800 353,200 353,600 354,000 354,400
hypothetical protein
alanine--tRNA ligase
group II intron reverse transcriptase/maturase
357,600 358,200 358,800 359,400 360,000 360,600 361,200 361,800 362,400
TruB family pseudouridylate synthase (N terminal ...
group II intron reverse transcriptase/maturase
reverse transcriptase domain-containing protein
hypothetical protein
378,000 378,400 378,800 379,200 379,600 380,000 380,400 380,800 381,200
FPG-type domain-containing protein
Retron-type reverse transcriptase
(pseudo) Group II intron reverse transcriptase/ma...
hypothetical protein
reverse transcriptase domain-containing protein
416,800 417,200 417,600 418,000 418,400 418,800 419,200 419,600 420,000
Group II intron reverse transcriptase/maturase
lipoate--protein ligase
molecular chaperone DnaJ
426,500 427,000 427,500 428,000 428,500 429,000 429,500 430,000 430,500
Retron-type reverse transcriptase
citrate lyase acyl carrier protein
Citrate lyase subunit beta
446,500 446,800 447,100 447,400 447,700 448,000 448,300 448,600 448,900 449,200
triphosphoribosyl-dephospho-CoA synthase
group II intron reverse transcriptase/maturase
Malate/citrate symporter
Retron-type reverse transcriptase
449,500 450,000 450,500 451,000 451,500 452,500 453,000 453,500 454,000 454,500
(pseudo) reverse transcriptase domain-containing ...
group II intron reverse transcriptase/maturase
tRNA-Lys(ttt)
GNAT family N-acetyltransferase
475,900 476,200 476,500 476,800 477,100 477,400 477,700 478,000 478,300 478,600
group II intron reverse transcriptase/maturase
Retron-type reverse transcriptase
Branched-chain alpha-keto acid dehydrogenase subu...
dihydrolipoyl dehydrogenase
515,000 515,500 516,000 516,500 517,000 517,500 518,000 518,500 519,000 519,500
group II intron reverse transcriptase/maturase
Group II intron reverse transcriptase/maturase
phosphatidate cytidylyltransferase
reverse transcriptase domain-containing protein
50S ribosomal protein L28
551,400 552,000 552,600 553,200 553,800 554,400 555,000 555,600 556,200 556,800
Group II intron reverse transcriptase/maturase
50S ribosomal protein L28
Bax inhibitor-1/YccA family protein
556,900 557,200 557,500 557,800 558,100 558,400 558,700 559,000 559,300
metallopeptidase TldD-related protein
hypothetical protein
group II intron reverse transcriptase/maturase
566,000 566,400 566,800 567,200 567,600 568,000 568,400 568,800 569,200 569,600
Retron-type reverse transcriptase
hypothetical protein
group II intron reverse transcriptase/maturase
reverse transcriptase domain-containing protein
hypothetical protein
569,200 569,600 570,000 570,400 570,800 571,200 571,600 572,000 572,400
Retron-type reverse transcriptase
reverse transcriptase domain-containing protein
(pseudo) reverse transcriptase domain-containing ...
Peptidase C69
ATPase
572,000 572,800 573,600 574,400 575,200 576,000 576,800 577,600 578,400 579,200
Retron-type reverse transcriptase
hypothetical protein
hypothetical protein
581,000 581,200 581,400 581,600 581,800 582,000 582,200 582,400 582,600
group II intron reverse transcriptase/maturase
hypothetical protein
hypothetical protein
600,000 601,500 603,000 604,500 606,000 607,500 609,000 610,500

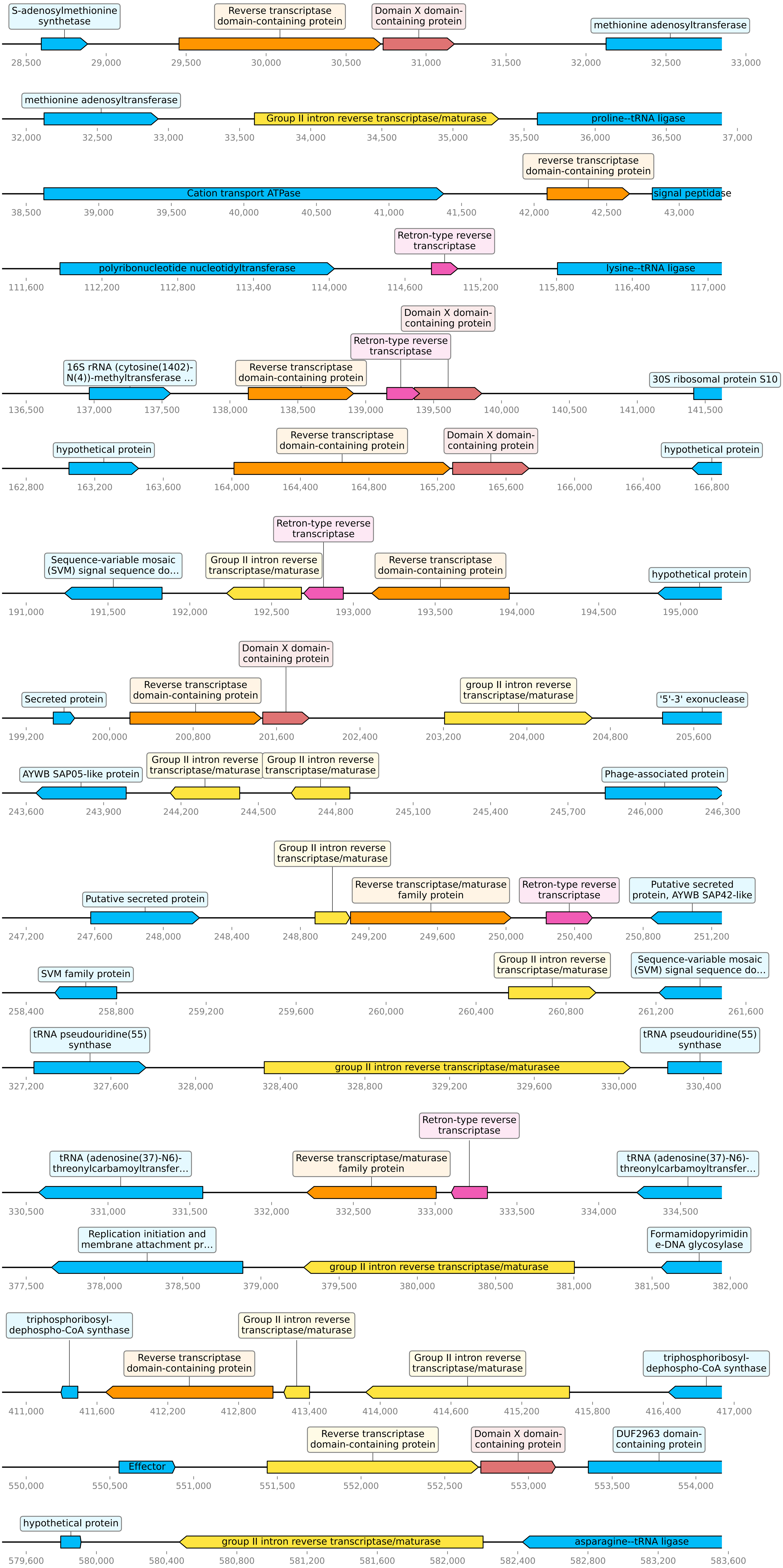
S-adenosylmethionine synthetase
Reverse transcriptase domain-containing protein
Domain X domain-containing protein
methionine adenosyltransferase
28,500 29,000 29,500 30,000 30,500 31,000 31,500 32,000 32,500 33,000
methionine adenosyltransferase
Group II intron reverse transcriptase/maturase
proline--tRNA ligase
32,000 32,500 33,000 33,500 34,000 34,500 35,000 35,500 36,000 36,500 37,000
reverse transcriptase domain-containing protein
Cation transport ATPase
signal peptidase
38,500 39,000 39,500 40,000 40,500 41,000 41,500 42,000 42,500 43,000
Retron-type reverse transcriptase
polyribonucleotide nucleotidyltransferase
lysine--tRNA ligase
111,600 112,200 112,800 113,400 114,000 114,600 115,200 115,800 116,400 117,000
Domain X domain-containing protein
Retron-type reverse transcriptase
16S rRNA (cytosine(1402)-N(4))-methyltransferase ...
Reverse transcriptase domain-containing protein
30S ribosomal protein S10
136,500 137,000 137,500 138,000 138,500 139,000 139,500 140,000 140,500 141,000 141,500
Reverse transcriptase domain-containing protein
Domain X domain-containing protein
hypothetical protein
hypothetical protein
162,800 163,200 163,600 164,000 164,400 164,800 165,200 165,600 166,000 166,400 166,800
Retron-type reverse transcriptase
Sequence-variable mosaic (SVM) signal sequence do...
Group II intron reverse transcriptase/maturase
Reverse transcriptase domain-containing protein
hypothetical protein
191,000 191,500 192,000 192,500 193,000 193,500 194,000 194,500 195,000
Domain X domain-containing protein
Secreted protein
Reverse transcriptase domain-containing protein
group II intron reverse transcriptase/maturase
'5'-3' exonuclease
199,200 200,000 200,800 201,600 202,400 203,200 204,000 204,800 205,600
AYWB SAP05-like protein
Group II intron reverse transcriptase/maturase
Group II intron reverse transcriptase/maturase
Phage-associated protein
243,600 243,900 244,200 244,500 244,800 245,100 245,400 245,700 246,000 246,300
Group II intron reverse transcriptase/maturase
Putative secreted protein
Reverse transcriptase/maturase family protein
Retron-type reverse transcriptase
Putative secreted protein, AYWB SAP42-like
247,200 247,600 248,000 248,400 248,800 249,200 249,600 250,000 250,400 250,800 251,200
SVM family protein
Group II intron reverse transcriptase/maturase
Sequence-variable mosaic (SVM) signal sequence do...
258,400 258,800 259,200 259,600 260,000 260,400 260,800 261,200 261,600
tRNA pseudouridine(55) synthase
group II intron reverse transcriptase/maturasee
tRNA pseudouridine(55) synthase
327,200 327,600 328,000 328,400 328,800 329,200 329,600 330,000 330,400
Retron-type reverse transcriptase
tRNA (adenosine(37)-N6)-threonylcarbamoyltransfer...
Reverse transcriptase/maturase family protein
tRNA (adenosine(37)-N6)-threonylcarbamoyltransfer...
330,500 331,000 331,500 332,000 332,500 333,000 333,500 334,000 334,500
Replication initiation and membrane attachment pr...
group II intron reverse transcriptase/maturase
Formamidopyrimidin e-DNA glycosylase
377,500 378,000 378,500 379,000 379,500 380,000 380,500 381,000 381,500 382,000
triphosphoribosyl-dephospho-CoA synthase
Group II intron reverse transcriptase/maturase
Reverse transcriptase domain-containing protein
Group II intron reverse transcriptase/maturase
triphosphoribosyl-dephospho-CoA synthase
411,000 411,600 412,200 412,800 413,400 414,000 414,600 415,200 415,800 416,400 417,000
Reverse transcriptase domain-containing protein
Domain X domain-containing protein
DUF2963 domain-containing protein
Effector
550,000 550,500 551,000 551,500 552,000 552,500 553,000 553,500 554,000
hypothetical protein
group II intron reverse transcriptase/maturase
asparagine--tRNA ligase
579,600 580,000 580,400 580,800 581,200 581,600 582,000 582,400 582,800 583,200 583,600

Fig. S4. Domain architecture of Bakta-predicted group II intron-encoded proteins in PR08 (A) and PR34 (B). In PR08 (A), several reverse transcriptase (RT) ORFs exhibit split configurations where RT and maturase (Domain X) domains are encoded by consecutive but separate genes, with some RTs lacking adjacent Domain X. Upstream intergenic regions (~500–600 bp) may represent partial intron RNA remnants. In PR34 (B), all identified intron ORFs are intact, harboring both RT and Domain X within a single ORF, although a few pseudo-RT ORF sequences show disrupted motifs.
