# Supplementary material for: Genomic landscape reveals the dominance of self-catalytic, high-copy group II introns in PMU-deficient complete genomes of PWB phytoplasmas
Source: J Microbiol. 2026 Mar 19;64(4):e2511004. doi: 10.71150/jm.2511004 (PMC13577128; doi:10.71150/jm.2511004)
Supplement: Supplementary file 5 [file jm-2511004-Supplementary-Table-S1.pdf]

Table S1. Classified intron-encoded proteins identified in '*Ca.* P. australasiaticum' strain PR08. Bakta annotation predicted 28 intron-encoded ORFs in PR08, including intact, split, and degenerate loci containing reverse transcriptase or maturase domains. Following BLASTp-based classification and redundancy filtering against a curated intron reference database, 15 non-redundant, high-confidence intron loci were retained and are listed here.

Query accession	Subject (PR08) accession	% Iden	% Q. Cov.	E-value	Bit score	RT class	Top hit organism
NP_049088.1	NKBAFF_0029	32.6	59	1.11E-68	230	ML	*Novosphingobium aromaticivorans*
AFL77071.1	NKBAFF_0032	33.1	99	6.68E-93	298	ML	*Alistipes finegoldii*
NP_049088.1	NKBAFF_0124	39.0	31	5.24E-53	183	ML	*Novosphingobium aromaticivorans*
NP_049088.1	NKBAFF_0164	32.6	59	2.89E-69	232	ML	*Novosphingobium aromaticivorans*
YP_001213018.1	NKBAFF_0186	43.9	48	8.69E-49	168	g6	*Pelotomaculum thermopropionicum*
NP_049088.1	NKBAFF_0191	31.6	59	3.80E-68	229	ML	*Novosphingobium aromaticivorans*
YP_002529852.1	NKBAFF_0194	31.1	82	5.74E-72	240	ML	*Bacillus cereus*
AAM99468.1	NKBAFF_0238	31.1	54	2.47E-37	139	UNCL	*Streptococcus agalactiae*
AFL77071.1	NKBAFF_0308	31.3	99	6.43E-88	285	ML	*Alistipes finegoldii*
YP_002540888.1	NKBAFF_0311	33.2	47	8.02E-41	148	A	*Agrobacterium radiobacter*
CBK76313.1	NKBAFF_0347	31.6	99	2.24E-86	281	ML	*Clostridium cf. saccharolyticum*
YP_002529852.1	NKBAFF_0373	32.9	82	1.31E-76	252	ML	*Bacillus cereus*
NP_049088.1	NKBAFF_0375	31.4	90	4.54E-92	296	ML	*Novosphingobium aromaticivorans*
NP_049088.1	NKBAFF_0495	32.9	59	1.80E-69	233	ML	*Novosphingobium aromaticivorans*
YP_002529852.1	NKBAFF_0517	33.5	93	1.07E-91	295	ML	*Bacillus cereus*

UNCL: Unclassified
