# Supplementary material for: Genomic landscape reveals the dominance of self-catalytic, high-copy group II introns in PMU-deficient complete genomes of PWB phytoplasmas
Source: J Microbiol. 2026 Mar 19;64(4):e2511004. doi: 10.71150/jm.2511004 (PMC13577128; doi:10.71150/jm.2511004)
Supplement: Supplementary file 6 [file jm-2511004-Supplementary-Table-S2.pdf]

Table S2. Classified intron-encoded proteins identified in '*Ca.* P. asiaticum' strain PR34. Bakta annotation predicted 52 intron-encoded ORFs in PR34, encompassing intact, split, and partially degenerate loci. After BLASTp-based classification and redundancy collapse against a curated intron reference database, 14 non-redundant, high-confidence intron loci were retained and are presented in this table.

Query accession	Subject (PR34) accession	% Iden	% Q. Cov.	E-value	Bit score	RT class	Top hit organism
YP_003691715.1	IGNLFN_0071	42.1	40	3.40E-47	165	C	*Desulfovibrio alkaliphilus*
YP_002529852.1	IGNLFN_0269	33.3	66	1.05E-58	202	ML	*Bacillus cereus*
YP_002529852.1	IGNLFN_0280	33.9	70	1.26E-68	230	ML	*Bacillus cereus*
NP_049088.1	IGNLFN_0309	31.6	91	1.82E-93	300	ML	*Novosphingobium aromaticivorans*
NP_049088.1	IGNLFN_0313	31.5	91	2.05E-92	298	ML	*Novosphingobium aromaticivorans*
NP_049088.1	IGNLFN_0317	32.5	90	1.51E-90	293	ML	*Novosphingobium aromaticivorans*
NP_049088.1	IGNLFN_0319	32.9	91	4.89E-94	302	ML	*Novosphingobium aromaticivorans*
AAP70045.1	IGNLFN_0368	35.6	76	7.08E-80	259	ML	*Lactococcus lactis*
NP_049088.1	IGNLFN_0377	39.7	31	6.89E-53	185	ML	*Novosphingobium aromaticivorans*
NP_049088.1	IGNLFN_0396	32.1	91	1.43E-94	303	ML	*Novosphingobium aromaticivorans*
YP_002125013.1	IGNLFN_0426	30.6	31	1.26E-07	52.8	g6	*Alteromonas macleodii*
NP_049088.1	IGNLFN_0504	32.5	73	5.85E-81	265	ML	*Novosphingobium aromaticivorans*
YP_004264445.1	IGNLFN_0508	37.8	48	3.70E-56	193	ML	*Syntrophobotulus glycolicus*
AAP70045.1	IGNLFN_0537	33.9	94	1.76E-92	296	ML	*Lactococcus lactis*
